# Rising rates of starch degradation during daytime and trehalose 6-phosphate optimize carbon availability

**DOI:** 10.1093/plphys/kiac162

**Published:** 2022-04-18

**Authors:** Hirofumi Ishihara, Saleh Alseekh, Regina Feil, Pumi Perera, Gavin M George, Piotr Niedźwiecki, Stephanie Arrivault, Samuel C Zeeman, Alisdair R Fernie, John E Lunn, Alison M Smith, Mark Stitt

**Affiliations:** Max Planck Institute for Molecular Plant Physiology, Potsdam-Golm, 14476, Germany; Max Planck Institute for Molecular Plant Physiology, Potsdam-Golm, 14476, Germany; Center for Plant Systems Biology and Biotechnology, Plovdiv 4000, Bulgaria; Max Planck Institute for Molecular Plant Physiology, Potsdam-Golm, 14476, Germany; John Innes Centre, Norwich Research Park, Norwich, NR4 7UH, UK; Institute of Molecular Plant Biology, ETH Zürich, Zürich, Switzerland; Max Planck Institute for Molecular Plant Physiology, Potsdam-Golm, 14476, Germany; Max Planck Institute for Molecular Plant Physiology, Potsdam-Golm, 14476, Germany; Institute of Molecular Plant Biology, ETH Zürich, Zürich, Switzerland; Max Planck Institute for Molecular Plant Physiology, Potsdam-Golm, 14476, Germany; Center for Plant Systems Biology and Biotechnology, Plovdiv 4000, Bulgaria; Max Planck Institute for Molecular Plant Physiology, Potsdam-Golm, 14476, Germany; John Innes Centre, Norwich Research Park, Norwich, NR4 7UH, UK; Max Planck Institute for Molecular Plant Physiology, Potsdam-Golm, 14476, Germany

## Abstract

Many plants, including Arabidopsis (*Arabidopsis thaliana*), accumulate starch in the light and remobilize it to support maintenance and growth at night. Starch synthesis and degradation are usually viewed as temporally separate processes. Recently, we reported that starch is also degraded in the light. Degradation rates are generally low early in the day but rise with time. Here, we show that the rate of degradation in the light depends on time relative to dawn rather than dusk. We also show that degradation in the light is inhibited by trehalose 6-phosphate, a signal for sucrose availability. The observed responses of degradation in the light can be simulated by a skeletal model in which the rate of degradation is a function of starch content divided by time remaining until dawn. The fit is improved by extension to include feedback inhibition of starch degradation by trehalose 6-phosphate. We also investigate possible functions of simultaneous starch synthesis and degradation in the light, using empirically parameterized models and experimental approaches. The idea that this cycle buffers growth against falling rates of photosynthesis at twilight is supported by data showing that rates of protein and cell wall synthesis remain high during a simulated dusk twilight. Degradation of starch in the light may also counter over-accumulation of starch in long photoperiods and stabilize signaling around dusk. We conclude that starch degradation in the light is regulated by mechanisms similar to those that operate at night and is important for stabilizing carbon availability and signaling, thus optimizing growth in natural light conditions.

## Teaser

Starch degradation in the light is regulated by similar mechanisms to those operating at night, stabilizing carbon availability, and thereby optimizing growth in natural light conditions

## Introduction

Many plant species including Arabidopsis (*Arabidopsis thaliana*) accumulate a substantial part of their fixed carbon (C) as starch in the light to support maintenance and growth at night (Smith and Stitt, 2007; Stitt and Zeeman, 2012). The proportion of fixed C that is allocated to starch increases when C is in short supply, for example, when plants are grown in low irradiance or in short-day conditions (Smith and Stitt, 2007; Sulpice et al., 2014; Mengin et al., 2017). It is usually assumed that the rate of starch accumulation in the light can be understood solely in terms of the regulation of starch synthesis. There is, however, longstanding evidence that starch is sometimes degraded in the light (Stitt and Heldt, 1981; Fondy et al., 1989; Servaites et al., 1989; Lu et al., 2005; Weise et al., 2006; Thalmann et al., 2016). Recently, Fernandez et al. (2017) showed that the rate of starch degradation rises progressively with time in the light. Furthermore, if a decrease in irradiance is imposed early in the day the stimulation of starch degradation is minimal, whereas decreases in irradiance at progressively later times in the day result in progressively larger stimulations of starch degradation. These observations led to the proposal that, in natural light regimes, a rising rate of starch degradation buffers growth against the gradual decline in irradiance and photosynthesis during the dusk twilight. This study had two main goals. The first was to investigate whether the regulation of starch degradation in the light can be understood in terms of concepts that have been developed to understand how degradation is regulated in the dark. The second was to experimentally investigate the biological importance of starch degradation in the light

In conditions in which the C supply restricts growth, starch is remobilized during the night at a near-constant rate, set such that starch is almost but not completely exhausted at dawn (Smith and Stitt, 2007). Recent work in Arabidopsis has shown that this pattern is maintained across a wide range of growth conditions (Hädrich et al., 2012; Pyl et al., 2012; Sulpice et al., 2014; Mengin et al., 2017). Furthermore, this pattern is robust in the face of sudden changes in irradiance in the preceding light period, sudden changes in the duration of the preceding light period, sudden changes in night temperature, and sudden light breaks in the night (Graf et al., 2010; Pyl et al., 2012; Scialdone et al., 2013; Pilkington et al., 2015; Feike et al., 2016; Moraes et al., 2019). Adjustment of starch turnover to short- and long-term changes in the light environment maximizes growth by ensuring that fixed C is rapidly invested in new biomass whilst avoiding deleterious periods of C starvation in the later part of the night (Stitt and Zeeman, 2012; Ishihara et al., 2015).

The circadian clock plays a key role in pacing starch degradation to dawn (Graf et al., 2010; Graf and Smith, 2011; Scialdone et al., 2013; Flis et al., 2019; Webb et al., 2019). Scialdone et al. (2013) proposed that information about the amount of starch (S) and the anticipated time to dawn (T, a function of the circadian clock) is integrated via arithmetic division to set the rate of starch degradation (*R*_d_ = *S*/*T*). This model explains many observations, especially the robust timing of starch degradation to the coming dawn in the face of sudden perturbations (see above). It has also been proposed that starch turnover is directly or indirectly regulated by metabolic inputs that alter circadian clock gene expression and clock phase to maintain sugar homeostasis (Feugier and Satake, 2013; Dodd et al., 2014; Seki et al., 2017; Webb et al., 2019). Proposed metabolic inputs include SUGAR NON-FERMENTING-RELATED PROTEIN KINASE 1 mediated regulation of BASIC LEUCINE ZIPPER PROTEIN 63 (bZIP63; Frank et al., 2018). Loss-of-function mutants of bZIP63 show slightly premature exhaustion of starch and small changes in the phasing and abundance of some transcripts encoding proteins that are involved in starch degradation (Viana et al., 2021). In addition, phasing of transcripts for several starch degradation enzymes to the middle of the 24-h cycle may, via “translational coincidence” (i.e. as a consequence of higher translation rates during the day than at night), increase the capacity of the entire starch degradation pathway in long days when higher rates of starch degradation will be required (Seaton et al., 2018). It can be envisaged that C-limited plants require a complex and flexible multilayered network with partly redundant components in order both to pace starch degradation to dawn robustly across a wide range of growth conditions and to facilitate rapid adjustment of the rate of starch degradation in response to day-to-day fluctuations in conditions.

In conditions in which the C supply exceeds the capacity for growth, starch is not fully utilized during the night (Cheng et al., 1998; Grimmer et al., 1999; Hädrich et al., 2012; Stitt and Zeeman, 2012; Sulpice et al., 2014; Pilkington et al., 2015; Figueroa et al., 2016). This raises the possibility that sugar signaling resulting from a build-up of assimilates might act to slow starch degradation and prevent complete use of starch during the night. Whilst there is no clear evidence that the sugar-signal trehalose 6-phosphate (Tre6P) is involved in the regulation of starch synthesis (Lunn et al., 2014; Figueroa et al., 2016; Figueroa and Lunn, 2016), two independent approaches provide evidence for its involvement in the regulation of starch degradation. First, using inducible overexpression of *otsA*, a bacterial *TREHALOSE-6-PHOSPHATE SYNTHASE*, Martins et al. (2013) showed that an induced increase in Tre6P rapidly inhibits starch degradation, resulting in a decrease in the level of sucrose and other sugars compared to control plants. Second, the *sweet11;12* mutant, which is defective in sucrose efflux proteins that export sucrose from mesophyll cells (Chen et al., 2012; Chen, 2014), has elevated levels of sucrose and Tre6P and only partially mobilizes its starch at night (dos Anjos et al., 2018). Meta-analysis of the relationship between Tre6P and starch degradation in lines inducibly overexpressing *otsA* and in the *sweet11;12* mutant indicated that Tre6P slows degradation below the rate that would be required to exhaust starch at dawn (dos Anjos et al., 2018). Furthermore, the relationship between Tre6P levels and the proportion of starch degraded during the night was similar after a sudden induced increase in Tre6P and in response to a constitutive increase of Tre6P in the *sweet11;12* mutant, even though sucrose and other sugars fall in the former and rise in the latter. These observations point to a major role for Tre6P in feedback regulation of starch degradation in the dark in C-replete conditions.

To further study the phenomenon of starch degradation in the light, we first examined whether the timing of the onset of degradation in the light is related to dawn or to dusk. We also explored in depth our earlier observation that reductions in light intensity trigger starch degradation in a time-of-day-dependent manner (Fernandez et al., 2017); in particular, we investigated if Tre6P inhibits starch degradation in the light. Using these data, we asked whether and which aspects of starch degradation in the light can be predicted from a skeletal version of the arithmetic division equation, which was developed previously to describe the observed patterns of starch degradation at night (Scialdone et al., 2013). We then tested whether starch degradation in the light indeed allows C availability and growth to be maintained as the rate of photosynthesis declines toward dusk and as photosynthesis increases gradually after dawn.

## Results

### Kinetics of starch accumulation in short and long photoperiods

To investigate whether the onset of starch degradation in the light is related to the timing of dawn or dusk, we first compared the patterns of starch accumulation in a long light period in plants that had previously experienced either very long-day lengths or very short-day lengths. We reasoned that if anticipated time of dusk determined the timing of onset of degradation in the light, this timing would differ in the two sets of plants, whereas if the onset of degradation in the light were set by time elapsed since dawn, the timing of onset of degradation would be the same in the two sets of plants. To achieve this comparison, we grew one set of plants in 6-h photoperiod days and a second set in 18-h photoperiod days, both at 160 µmol m^−2^ s^−1^. The long-day plants were transferred to 80 µmol m^−2^ s^−1^ for 5 days before the experiment to ensure that they had low starch levels at the start of the experiment. On the day of the experiment, the short-day plants were transferred to continuous light at 90 µmol m^−2^ s^−1^. This was done to prevent accumulation of large amounts of starch during the subjective night. The long-day plants were illuminated at 50 µmol m^−2^ s^−1^.

**Figure 1:**
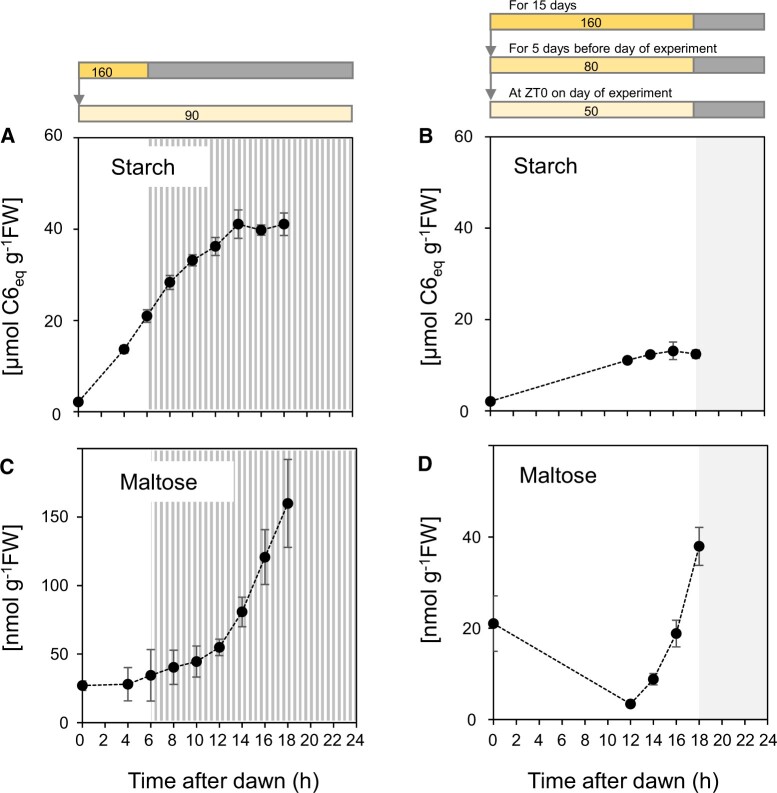
Temporal kinetics of starch accumulation and maltose content when plants are grown in a 6-h photoperiod and transferred to continuous low light, or are grown in an 18-h photoperiod. A and C, Plants were grown in a 6-h light/18-h dark cycle for 38 days with a light intensity of 160 µmol m^−2^ s^−1^ and then transferred at dawn to 90 µmol m^−2^ s^−1^ continuous light. The striped zone marks the subjective night. Error bars are 95% confidence limits (*n* = 5–6 except ZT0, *n* = 3) B and D, Plants were grown in an 18-h light/6-h dark cycle for 15 days with a light intensity of 160 µmol m^−2^ s^−1^, switched to 80 µmol m^−2^ s^−1^ for 5 days to reduce starch levels at dawn, and then harvested during an 18-h light period at 50 µmol m^−2^ s^−1^. The gray zone marks the night. Error bars are 95% confidence limits (n =5 except ZT0, n=4). A and B, Starch content. C and D, Maltose content. Underlying data are provided in Supplemental Data Sets 1 and 2.

In short-day plants after transfer to continuous light, starch accumulated in a near-linear manner until about ZT10 (10 h after dawn, 4 h after subjective dusk; “subjective” refers to when dusk occurred in the growth regime) before slowing and plateauing from about ZT14 onwards (Figure 1A; Supplemental Data Set 1, see Supplemental Table S1 for a list of all experiments, experimental design and accompanying datasets presented in this manuscript). Although as expected (Mengin et al., 2017), the absolute rate of starch accumulation was much lower in the long-day plants (Figure 1B; Supplemental Data Set 2), the pattern of change in starch content from ZT12 on was almost identical to that in short-day plants. These data are consistent with the onset of rapid starch degradation around ZT12, regardless of the length of light period previously experienced by the plants or the absolute rate of accumulation of starch. The robustness and reproducibility of these observations were confirmed by the similarities of patterns of starch accumulation in plants previously grown in 6-h or 12-h days (Fernandez et al., 2017) before transfer to continuous light, or in 18-h days (Supplemental Figure S1; Supplemental Data Set 3). These data support the idea that the onset of starch degradation is related to time elapsed since dawn, and is largely independent of the anticipated time of dusk.

### Response of starch accumulation to a decrease in irradiance

To examine further whether the onset of starch degradation is a function of time after dawn rather than anticipated time of dusk, we measured the impact of decreases in irradiance imposed at different times on the accumulation of starch. We showed previously for plants grown in 12-h days that the effect of a decrease in irradiance depended on when the decrease was imposed. A decrease at midday (ZT6) resulted in a reduced starch accumulation rate commensurate with the reduction in photosynthesis, whereas the same decrease after ZT9 resulted in a plateauing or even a decline in starch levels (Fernandez et al., 2017). These published data, while consistent with the triggering of starch degradation by decreases in irradiance late in the day, did not reveal whether this was related to the length of time elapsed since dawn, or to the anticipated timing of dusk.

To provide this information, we used short-day and long-day plants, grown as described in the previous section to ensure that starch levels were low at dawn on the day of the experiment. If the timing of onset of degradation is a function of time after dawn, a decrease in irradiance will trigger degradation beyond the same point after dawn regardless of the day length at which the plants were grown. If the onset of degradation is a function of the anticipated time of dusk, short-day and long-day plants will differ by several hours in the time beyond which a decrease in irradiance triggers degradation. During the day of the experiment, irradiance was decreased at various times from 160 to 90 µmol m^−2^ s^−1^ for short-day plants and from 80 to 50 µmol m^−2^ s^−1^ for long-day plants. These decreases, which were previously shown to cause a decline in the rate of photosynthesis of about 60% (Mengin et al., 2017), were imposed for 2–4 h, starting at times that were 2 or 4 h before anticipated dusk (ZT4 for short-day plants, ZT14 for long-day plants). Additionally, decreases were imposed about one-third of the way through the day for long-day plants (ZT6).

**Figure 2:**
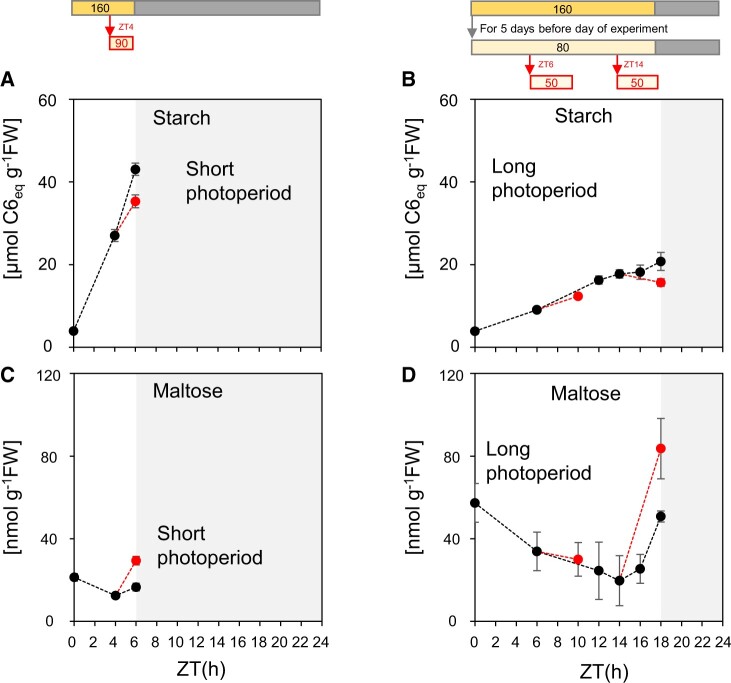
Response of starch content and maltose to a decrease in light intensity at different times in the light period in plants growing in a short photoperiod or a long photoperiod. A and C, Plants were grown in a 6-h light/18-h dark cycle at a light intensity of 160 µmol m^−2^ s^−1^ for 38 days. On the 39th day after sowing, plants were left at 160 µmol m^−2^ s^−1^ for 6 h, or were illuminated at 160 µmol m^−2^ s^−1^ until ZT4 and then transferred to 90 µmol m^−2^ s^−1^ from ZT4 to ZT6. Error bars are 95% confidence limits (*n* = 6). B and D, Plants were grown in an 18-h light/6-h dark cycle for 15 days at a light intensity of 160 µmol m^−2^ s^−1^, transferred to 80 µmol m^−2^ s^−1^ for 5 days to reduce dawn starch levels, then on the 21st day after sowing were left at 80 µmol m^−2^ s^−1^ or were initially illuminated at 80 µmol m^−2^ s^−1^ and then transferred to 50 µmol m^−2^ s^−1^ between ZT6 and ZT10, or between ZT14 and ZT18. Error bars are 95% confidence limits (*n* > 5). A and B Starch content. C and D, Maltose content. Sucrose and Tre6P levels from this experiment are plotted in Supplemental Figure S5. More underlying data are provided in Supplemental Data Sets 4 and 5.

The effect of these decreases in irradiance on starch accumulation depended on the time at which they occurred relative to dawn (ZT0), but not relative to subjective dusk. After irradiance decreases at ZT4 in short-day plants (Figure 2A; Supplemental Data Set 4) and at ZT6 in long-day plants (Figure 2B; Supplemental Data Set 5), starch accumulation slowed down to an extent that was roughly commensurate with the reduced rate of photosynthesis. After an irradiance decrease at ZT14 in long-day plants, starch content declined (Figure 2B). These data are consistent with the response of starch degradation to decreased light being weak at early times in the 24-h cycle and becoming stronger beyond ZT10. They do not support the notion that the onset of degradation is related to the timing of dusk; if this were the case, the responses of starch content to decreases in irradiance at ZT4 in short-day plants and ZT14 in long-day plants should be the same since both are shortly before an anticipated dusk.

### Labeling of starch during ^13^CO_2_ pulses at different times in the 24-h cycle

As an independent means of detecting starch degradation in long-day and short-day plants, we imposed decreases in irradiance as described above and simultaneously supplied a 2–4 h pulse of ^13^CO_2_. The total amount of starch that accumulated during the pulse was compared with the amount of ^13^C that accumulated in starch. Similar increases in ^13^C-starch and total starch (i.e. ^13^C- plus ^12^C-starch) during the pulse would be consistent with the idea that preexisting starch is not being degraded. A larger increase in ^13^C-starch than in total starch (i.e. a decrease in the amount of ^12^C starch) would show that some of the preexisting starch is being degraded. This is plausible given the recent demonstration that Arabidopsis leaf starch granules grow anisotropically (Bürgy et al., 2021), such that parts of the granule surface could be elaborated with newly fixed C, while other parts of the granule surface are degraded, releasing preexisting starch. Nevertheless, the results of these experiments must be interpreted with caution because cycling of ^13^C out of nascent glucans or newly synthesized starch would lead to underestimation of the rates of both synthesis and degradation (see Fernandez et al., 2017 for more extensive discussion)

**Figure 3:**
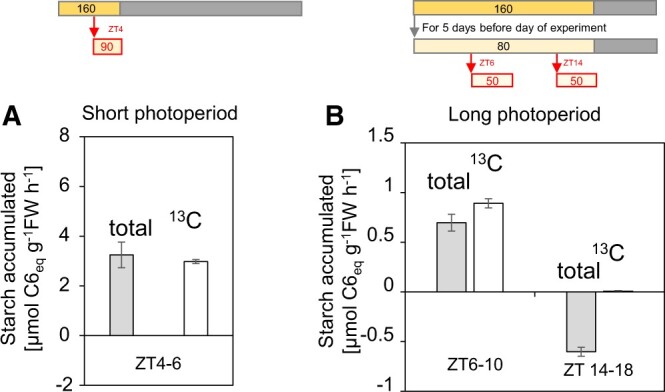
^13^CO_2_ labeling of starch during a decrease in irradiance at different times in the light period. Labeling was carried out as follows: (A) Before dusk in short day-grown plants. Plants were grown in a 6-h light/18-h dark cycle for 38 days with light intensity of 160 µmol m^−2^ s^−1^. On the 39th day, plants were illuminated at 160 µmol m^−2^ s^−1^ until ZT4 then transferred to 90 µmol m^−2^ s^−1^ from ZT 4 to ZT6 (starch level is shown in Figure 2A). 13CO_2_ was supplied between ZT4 and ZT6. B, In the middle of the light period and before dusk in long-day-grown plants. Plants were grown in an 18-h light, 6-h dark cycle at 160 µmol m^−2^ s^−1^ for 15 days followed by 5 days at 80 µmol m^−2^ s^−1^, and on the 21st day were supplied with ^13^CO_2_ between ZT7 and ZT10 or between ZT14 and ZT18. Starch was isolated from plants harvested at the start and end of each labeling interval. It was hydrolyzed to glucose and analyzed by GC–MS to determine the amounts of ^13^C-labeled starch and ^12^C-labeled starch. The displays show the estimated rates of accumulation of total starch (^12^C plus ^13^C; gray bars) and ^13^C starch (white bars). The results are given as mean values (for (A), *n* = 8 for ZT4 and *n *= 6 for ZT6; for (B), *n* = 4–5). Error bars were calculated using the standard error of the mean for Gaussian error propagation. Underlying data are provided in Supplemental Data Sets 4 and 6.

When ^13^CO_2_ was supplied to short-day plants at the same time as a decrease in irradiance at ZT4, or to long-day plants at the same time as a decrease in irradiance at ZT6, ^13^C-starch and total starch increased to similar extents over the next 2 h (Figure 3A; Supplemental Figure S2). However, when ^13^CO_2_ was supplied to long-day plants at the same time as a decrease in irradiance at ZT14, the changes in ^13^C starch and ^12^C starch over the next 4 h were very different from each other. Whereas little or no ^13^C starch accumulated, the total amount of starch actually declined (Figure 3B; Supplemental Figure S3A; Supplemental Data Set 6). These data are consistent with substantial degradation of preexisting starch after a decrease in irradiance at ZT14 in long-day plants, but not at ZT4 or ZT6 in short- and long-day plants, respectively.

### Maltose content

Maltose is the first major product of leaf starch degradation (Stitt and Zeeman, 2012), and maltose levels are usually positively related to the rate of starch degradation (Lu et al., 2005). Maltose acts as a buffer between the irreversible reactions of its production from starch and its use via the equilibrium reactions catalyzed in the cytosol by DISPROPORTIONATING ENZYME 2 (DPE2) and ALPHA-GLUCAN PHOSPHORYLASE 2 (Ruzanski et al., 2013). Whilst maltose content does not provide quantitative information about the rate of starch degradation, it does provide useful qualitative information that is independent of measurements of starch content per se.

Irrespective of the photoperiod in which plants had been grown, after transfer to continuous light maltose levels started to rise markedly from about 12 h after dawn (Figures 1, C, D and 2, D; Supplemental Figure S1B). When a sudden decrease in irradiance was imposed at ZT4 in short-day and ZT6 in long-day plants maltose rose only slightly (Figure 2, C and D). In contrast, maltose rose sharply when a sudden decrease in irradiance was imposed at ZT14 in long-day plants (Figure 2D). These changes in maltose content provide qualitative support for the two main conclusions drawn from changes in starch content; that starch degradation rate in the light increases with time after dawn and that a sudden decrease in irradiance leads to only a slight stimulation of starch degradation when imposed early in the 24-h cycle but a large stimulation when it is imposed later in the 24-h cycle. Furthermore, maltose levels were highest in the 6-h-day grown plants and lower in the 12-h and especially the 18-h grown plants (Supplemental Figure S1B), mirroring the absolute amount of starch accumulated in these conditions (Supplemental Figure S1A). It might also be noted that, relative to levels in the rest of the 24-h cycle, maltose levels are low at dawn in short-day plants (Figure 1C; Supplemental Figure 1B) but quite high in long-day plants (Figures 1, D and 2, D) (see below for more data and discussion).

### A skeletal arithmetic division equation largely predicts the pattern of starch degradation in the light

The arithmetic division model (Scialdone et al., 2013) was formulated to explain the regulation of starch degradation at night, and is centered on the idea that the rate of degradation, R_d_, is a function of the amount of starch (S) divided by the remaining time to dawn (T). The finding that the onset of rapid starch degradation in the light depends on time relative to dawn, rather than dusk, prompted us to ask if a skeletal form of the arithmetic division equation can also be used to predict changes in starch content after transfer to continuous light. The following data analysis is independent of the molecular models developed by Scialdone et al. (2013) to explore how such an arithmetic division might be performed; it simply asks if the skeletal formula *R*_d_ = *S/T* that describes the observed responses at night can also be used to predict temporal changes in starch content in the light. With time in the light, starch content rises, while the time until the next dawn decreases. This should give rise to an increasing rate of starch degradation with time, if the model holds in these conditions. Assuming that the rate of starch synthesis is constant in the light (see below for data supporting this assumption), an increasing rate of degradation with time could result in a progressive shift from net starch accumulation to net starch mobilization. These expectations are in line with our measurements of starch and maltose, which point to a gradual rise in the starch degradation rate with time in the light (decreasing time until the next dawn) and are thus qualitatively consistent with the response predicted by the arithmetic division equation.

**Figure 4:**
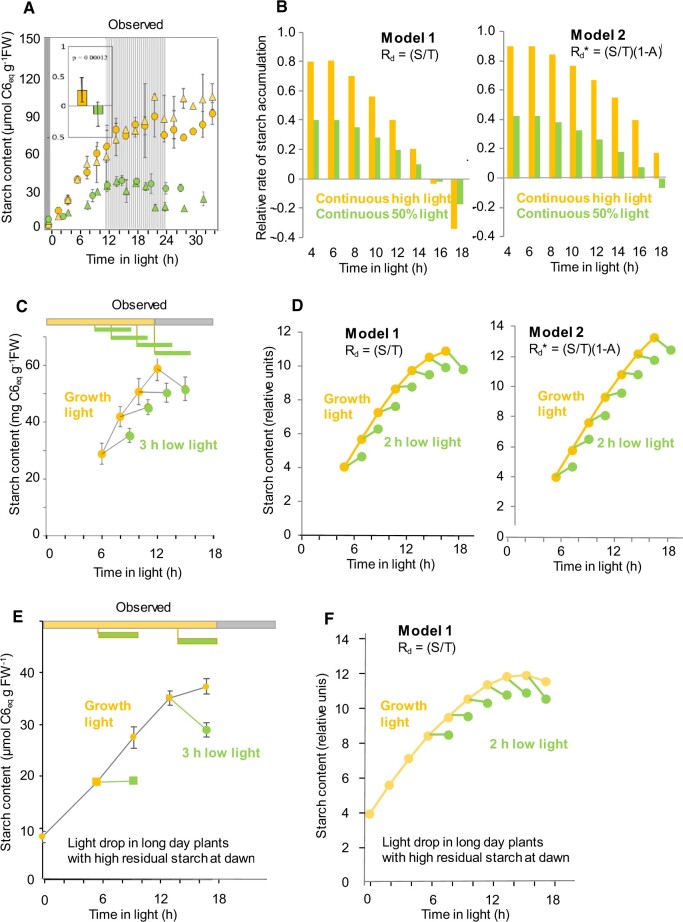
Observed and simulated response of the net rate of starch accumulation or mobilization with increasing duration of the light period, or in response to a sudden decrease in light intensity. The simulations are described in “Materials and methods” and Supplemental calculation S2). Briefly, two models are used to estimate the rate of starch degradation. In Model 1, the rate of starch degradation *R*_d_ = *S/T*, where *S* is the amount of starch and *T* is the remaining time to dawn. In Model 2, the rate of starch degradation R_d_* = (*S/T*)(1-A) where *A* is a nominal term for a rate of photosynthesis-dependent inhibition of starch degradation, and is assumed in molecular terms to represent inhibition by Tre6P. The rate of starch synthesis, *R_s_*, is assumed to be constant. Units are relative. *R_s_* was set as 1 and 0.5 relative units h^−1^, respectively, in growth irradiance and under a lower irradiance that led ∼50% inhibition of photosynthesis. Rates of starch degradation and change in net starch content are given as a fraction of *R_s_* in growth irradiance. A, Observed response of net starch accumulation after transferring plants previously grown in a 12-h light/12-h dark cycle at dawn to continuous light at growth irradiance or continuous low light. The left-hand display shows the time course of starch accumulation in one experiment after transferring wild-type Col-0 (circles) or Ws-2 (triangles) from a 12-light (150 µmol m^−2^ s^−1^)/12-h dark cycle to continuous light at growth irradiance (150 µmol m^−2^ s^−1^) or a lower irradiance (90 µmol m^−2^ s^−1^) that would approximately half the rate of photosynthesis. Results are plotted as mean ± sd of measurements on two pots, each of which contained five individual plants (see Fernandez et al., 2017). The insert display is a combined analysis of this and 11 further experiments. It shows (as a fraction of the rate in the subjective day) the rate of starch accumulation in the subjective night after transfer to continuous light at an intensity similar to or higher than growth irradiance or continuous low light. The experiments and time intervals used for the calculation are listed in Supplemental calculation S1. A negative value indicates net mobilization. The result is plotted as mean ± sd (*n *= 13 separate experiments). The response was significantly different between growth light and low light (*P* < 0.0001, two-tailed *t* test with equal variance). B, Simulated net rate of starch accumulation at successive 2-h intervals through the light period after transferring plants grown in a 12-h light/12-h dark cycle to continuous light at growth irradiance or a lower light intensity that halves the rate of photosynthesis. Negative values indicate net mobilization. The simulations use either Model 1 (left-hand display) or Model 2 (right-hand display). Starch content at dawn was assumed to be negligible (see Figure 4A). C, Observed response of starch content to a 3-h light drop from 150 µmol m^−2^ s^−1^ to 90 µmol m^−2^ s^−1^ at different times in the light period. The data (mean ± sd of measurements on six rosettes) are taken from Fernandez et al. (2017), see also Figure 2, A and C and Supplemental Figure S4, A and C. D, Simulated response of starch content with time in growth irradiance (yellow) and the change in starch content following a 50% decrease in light intensity for 2 h (green) at different times during the light period. The simulations use either Model 1 (left-hand display) or Model 2 (right-hand display). Starch content at dawn was set as negligible (see Figure 4A). E, Observed change in starch content after illumination of plants grown in an 18-h light (160 µmol m^−2^ s^−1^)/6-h dark cycle at growth irradiance and after decreasing irradiance to 90 µmol m^−2^ s^−1^ between ZT6–ZT10, or between ZT14–ZT18 ( (mean ± 95% confidence limits, *n* = 4). Note that the plants started the day with a high dawn starch content because they were grown in a long photoperiod at high light until the day of the experiment. Underlying data are provided in Supplemental Data Set 8. F, Simulated change in starch content with time in an 18-h photoperiod and following a 50% decrease in light intensity for 2 h at different times during the light period. The simulation uses Model 1 and is for plants grown in an 18-h photoperiod. A dawn starch content was selected with which the simulation generated an approximately three-fold higher value at the peak at ZT14, resembling the relation in the empirical data set in (E).

To provide a more rigorous test of the ability of the skeletal arithmetic division equation to predict the observed changes in starch content in the light, we collated data on starch content from many experiments in which plants were transferred at dawn to continuous light at either 160 or 90 µmol m^−2^ s^−1^. We collated data because there is experiment-to-experiment variation in the response of starch content in continuous light; this may be due partly to biological noise and to differences in the metabolic status of the plants between experiments. One such experiment with Arabidopsis accessions Col-0 and Ws-2 is shown in the left-hand display of Figure 4A (data from Fernandez et al., 2017). In this experiment, net starch accumulation slowed down as the subjective night progressed and by about ZT18 starch content had almost plateaued in continuous high light and was declining in continuous low light. We analyzed the two experiments of Figure 4A together with a further 11 experiments in which plants were transferred to continuous high light and 11 experiments in which plants were transferred to continuous low light (Supplemental Calculation S1; data from Fernandez et al. (2017) and this study). In continuous high light, the rate of accumulation in the subjective night slowed to 26% ± 22% (mean ± sd) of that in the subjective day, whereas in continuous low light the rate slowed to −15% ± 22% of that during the subjective day (i.e. on average, net starch mobilization). The difference between the two responses was significant (*t* test, *P* < 0.0001, two-tailed *t* test with equal variance) (see Supplemental Calculation S1 for the time intervals used for the calculations).

A simulation using the skeletal arithmetic division equation (*R*_d_ = *S/T*) (Supplemental Calculation S2, Model 1) predicted the observed gradual slowing down of net starch accumulation with time in the light (Figure 4B, left-hand display). There was, however, a clear discrepancy between the simulated and observed values. The simulation predicted faster net starch mobilization in the later part of the subjective night in high light than in low light (Figure 4B); this prediction is a consequence of the faster starch accumulation and hence higher starch content at any given time in high light than in low light. This prediction differs from the observed responses, in which starch accumulation usually only slows down or plateaus in high light, whereas it reverses in low light (Figure 4A).

As already mentioned, a decrease in light intensity early in the 24-h cycle leads to a slowing of starch accumulation in line with the decrease in the rate of photosynthesis, whereas a decrease in light intensity later in the cycle leads to net starch mobilization (see Figure 4C for a typical response taken from Fernandez et al., 2017, also Figure 2 and below). The skeletal arithmetic division equation correctly predicted this time-of-day-dependent response to a decrease in light intensity (Figure 4D, left-hand display).

The arithmetic division equation further predicts that the response of starch accumulation to a decrease in light intensity early in the day will depend upon the amount of starch remaining at dawn. If starch is almost entirely depleted by dawn, starch content in the first part of the day will be low and so the rate of degradation following a decrease in light intensity will be low. If night-time starch degradation is incomplete, starch content in the first part of the day will be relatively high, resulting in a relatively high rate of degradation following a decrease in light intensity. To test this prediction, we grew plants in an 18-h photoperiod at 160-µmol m^−2^ s^−1^ irradiance, conditions in which substantial starch remained at dawn (Figure 4E; see also Sulpice et al., 2014). After 6 h in the light, some plants were subjected to a decrease in light intensity to 90 µmol m^−2^ s^−1^. The decrease resulted in the cessation of starch accumulation (Figure 4E, Supplemental Data Set 7), in marked contrast to the continued net accumulation of starch when a decrease in light intensity was imposed at ZT6 on plants with low starch contents at dawn (Figures 2 and 4C, see also below and Fernandez et al., 2017). There was a good qualitative match between these experimentally observed responses and those simulated using the skeletal arithmetic division equation (compare Figure 4, E with Figure 4, F).

Taken together, the skeletal arithmetic division equation can reproduce many features of starch degradation in the light. However, it overestimates the rate of starch degradation in high light.

### Starch degradation in the light is influenced by metabolic signals rather than signals related to light intensity

We considered two possible factors that might cause the discrepancy between the observed changes in the net starch accumulation with time in high light and those predicted by the arithmetic division equation. First, the rate of starch degradation might be under gated control by light signaling, and second it might be influenced by changes in leaf metabolism in the light that are related directly or indirectly to photosynthetic C fixation.

**Figure 5:**
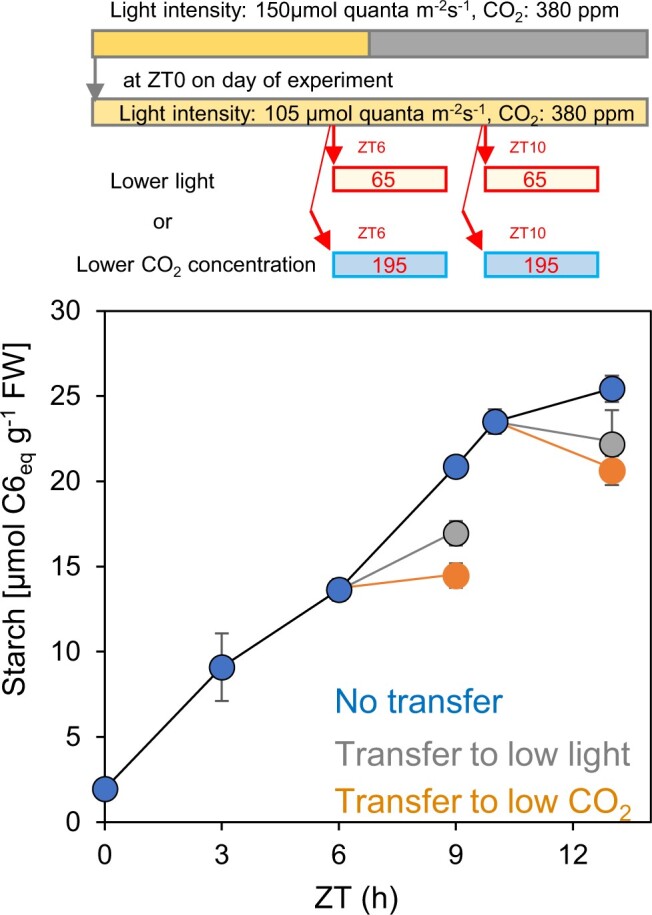
Response of starch content to a drop in light intensity or CO_2_ concentration after different times in the light. Plants were grown in 12-h light/12-h dark at 150 μmol quanta m^−2^ s^−1^ and 380 ppm CO_2_ for 24 days. On the day of the experiment, the plants were illuminated at 105 µmol m^−2^ s^−1^, 380 ppm CO_2_. Some were transferred to lower light (65 µmol m^−2^ s^−1^) or lower CO_2_ (195 ppm CO_2_) at ZT6 and harvested at ZT9. Others were transferred to lower light (65 µmol m^−2^ s^−1^) or lower CO_2_ (195 ppm CO_2_) at ZT10 and left in these conditions until harvest at ZT13. Control plants remained in 105 µmol m^−2^ s^−1^ and 380 ppm CO_2_ until ZT13. Blue symbols are starch levels of control plants; gray symbols are levels for plants transferred to low light; orange symbols are levels for plants transferred to low [CO_2_]. Values are means ± SE of measurements on 5–12 individual rosettes. Measurements of photosynthesis showing that the decrease in light intensity and in [CO_2_] caused similar reductions in the rate of photosynthesis are provided in Supplemental Figure S4.

To investigate whether the response of starch degradation to a decrease in light intensity is due to the change in light intensity per se or the resultant decrease in the rate of photosynthesis, we compared the impact of a decrease in light intensity with that of a decrease in CO_2_ concentration that caused the same decrease in the rate of photosynthesis (Supplemental Figure S4; Supplemental Data Set 8). Plants were subjected to decreases in either light intensity or CO_2_ concentration for 3 h from ZT6–ZT9 to from ZT10–ZT13. As expected, the decreases in light intensity resulted in a much greater reduction in starch accumulation at ZT10–ZT13 than at ZT6–ZT9. The same was true of the decreases in [CO_2_]; starch accumulation changed much more following a decrease at ZT10–ZT13 than at ZT6–ZT9 (Figure 5; Supplemental Figure S7). These data suggest strongly that the kinetics of starch accumulation with time are influenced by metabolic changes related to photosynthesis rather than by light signaling.

### Impact of an induced increase in Tre6P on starch degradation in the light

**Figure 6:**
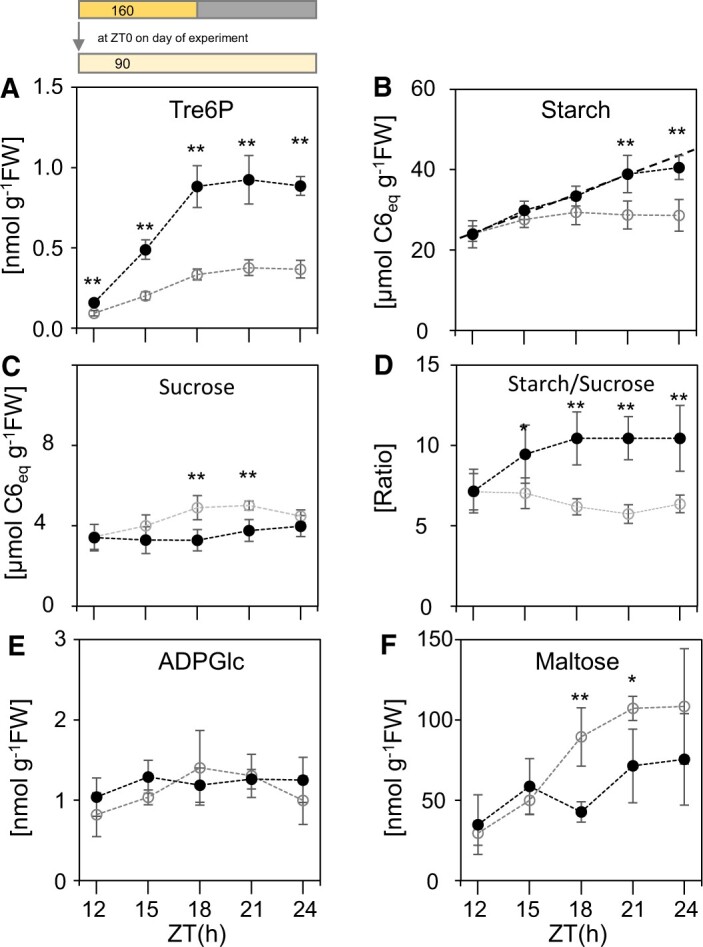
Impact of an induced increase in Tre6P on starch content and metabolite levels in the light. TPS29.2 and AlcR plants (solid symbols, TPS29.2; open symbols, AlcR) were grown in a 12-h light/12-h dark cycle at a light intensity of 160 µmol m^−2^ s^−1^ for 21 days and then transferred to continuous light at 90 µmol m^−2^ s^−1^. The plants were sprayed with 2% v/v ethanol at ZT10 and samples were harvested at 3-h intervals, starting at ZT12. A, Tre6P, (B) Starch (dotted line indicates starch content if the rate of accumulation between ZT0 and ZT12 were to have continued), (C) Sucrose, (D) Starch/sucrose ratio, (E) ADPGlc, and (F) maltose. Values are means ± 95% confidence interval (*n* = 5, each sample containing 5 individual rosettes). Asterisks indicate statistically significant differences between TPS29.2 and AlcR, Student’s *t* test: *P < 0.05, **P < 0.01. Underlying data are provided in Supplemental Data Set 9. Incidentally, the near-constant level of ADPGlc for up to 24 h in continuous light provides support for our assumption in the model underlying Figure 4 that the rate of starch synthesis rate does not decrease with time in the light. High or even slightly rising ADPGlc levels were also reported between ZT12 and ZT18 in earlier studies of plants growing in long-day conditions (Figueroa et al., 2016; Fünfgeld et al., 2021).

A possible candidate for a metabolic signal generated by changes in the rate of photosynthesis would be a change in levels of sucrose and the associated sucrose-signaling molecule Tre6P. Both sucrose levels and Tre6P levels increase with the rate of photosynthesis (Mengin et al., 2017) and with time in the light (Martins et al., 2013; Sulpice et al., 2014; Figueroa et al., 2016, Annunziata et al., 2017; Flis et al., 2019; see also Supplemental Figure S5 and below). It is already known that Tre6P inhibits starch degradation at night (Martins et al., 2013; dos Anjos et al., 2018). We previously used transgenic lines carrying a bacterial *trehalose-6-phosphate synthase* (*TPS*) gene under the control of the ethanol-inducible ALCOHOL DEHYDROGENASE REGULATOR (*AlcR*) promoter to show that elevation of Tre6P during the night results in a reduced rate of starch degradation (Martins et al., 2013). We used one of these transgenic lines (*TPS29.2*) to test whether elevation of Tre6P also inhibits starch degradation in the light. As a control we used *AlcR* lines that express the AlcR ethanol-binding transcription factor but do not carry the bacterial *tps* gene (Figure 6).

Plants were grown in a 12-h photoperiod at 160 µmol m^−2^ s^−1^ and transferred to continuous low light (90 µmol m^−2^ s^−1^) at ZT0, then sprayed with ethanol at ZT10 to induce AlcR protein and (in the case of *TPS29.2*) Tre6P production. Levels of Tre6P were significantly higher in *TPS29.2* than *AlcR* by ZT12, and up to 2.5-fold higher between ZT15 and ZT24 (Figure 6A; Supplemental Data Set 9). The magnitude of the induced increase in Tre6P resembled that seen in previous studies in which TPS was induced at the beginning of the light period (Figueroa et al., 2016) or the beginning of the night (Martins et al., 2013). Whereas the starch content in *AlcR* showed very little increase or even declined slightly after ZT15, the starch content of *TPS29.2* continued to rise (Figure 6B). This elevation of starch plus a reduced sucrose content in the *TPS29.2* line from ZT18 onwards (Figure 6C) resulted in starch:sucrose ratios that were almost twice as high in *TPS29.2* as in *AlcR* (Figure 6D). The different rates of starch accumulation were unlikely to be caused by differences in the rate of starch synthesis; the level of the dedicated starch synthesis substrate ADP-glucose was the same in *TPS29.2* and *AlcR* (Figure 6E). The 1.5- to 2-fold lower maltose content in *TPS29.2* compared to *AlcR* between ZT18 and ZT21 (Figure 6F) pointed to slower starch degradation in *TPS29.2* as the main reason for the difference in starch content. Together, these results are consistent with the idea that elevated Tre6P promotes starch accumulation by reducing the rate of starch degradation in the light.

**Figure 7:**
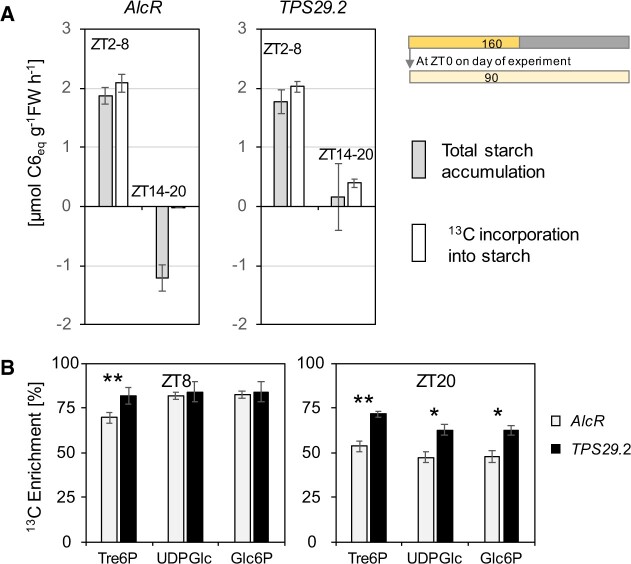
Impact of an induced increase in Tre6P on ^13^C labelling of starch and metabolites in the light. The experiment was conducted as in Figure 6, spraying with 2% v/v ethanol at ZT10 to induce an increase in Tre6P (see Figure 6 for the changes of Tre6P, total starch content and other metabolites). ^13^CO_2_ was supplied at ambient concentration (420 ppm) between ZT2 and ZT8 (before the induced increase in Tre6P) and between ZT14 and ZT20 (after the induced increase in Tre6P). A, Estimated rate of change in total starch content (gray bar) and rate of ^13^C incorporation into starch (open bars). The amounts of ^12^C starch and ^13^C-labeled starch at ZT2, ZT8, ZT14, and ZT20 that were used to estimate these rates are provided in Supplemental Figure S6. Error bars were calculated using the se of the mean for Gaussian error propagation. B, ^13^C enrichment in Tre6P, UDPGlc, and Glc6P in samples harvested at ZT8 and ZT20 (i.e. at the end of each 6-h pulse). Enrichment in Calvin–Benson cycle intermediates is typically about 80%, and is slightly lower in Tre6P and the metabolites involved in sucrose synthesis (Szecowka et al., 2013). Lower enrichment values indicate there is an additional influx of unlabeled C that does not derive from current C fixation. Values are means ± 95% confidence interval (*n* = 5, each sample containing five individual rosettes). Asterisks indicate statistically significant differences between *TPS29.2* and *AlcR*, Student’s *t* test: *P < 0.05, **P < 0.001) Underlying data are provided in Supplemental Figure S6 and Supplemental Data Set 9.

^13^CO_2_ labeling experiments were used to provide further information about the impact of Tre6P on starch degradation in the light (Figure 7; Supplemental Figure S6; Supplemental Data Set 10). *TPS29.2* and *AlcR* plants were moved to continuous low light as above, and ^13^CO_2_ was supplied for 6 h either between ZT2 and ZT8 (before induction with ethanol) or between ZT14 and ZT20 (after induction with ethanol). Between ZT2 and ZT8, total starch and ^13^C-starch accumulated in similar amounts in both lines (Figure 7A). Between ZT14 and ZT20, results for the two lines were very different. *AlcR* showed no incorporation of ^13^C into starch, and there was substantial loss of total starch (see also Figures 1 and 3; Supplemental Figures S2 and S3 and Fernandez et al., 2017). In contrast, *TPS29.2* showed incorporation of ^13^C into starch and further net accumulation of starch, although at rates lower than those seen between ZT2 and ZT8. These data are consistent with the expected occurrence of substantial starch degradation in the light during the latter part of the day but not early in the day, and also with inhibition by Tre6P of starch degradation in the light.

Further information consistent with starch degradation in the light and its inhibition by elevation of Tre6P in the transgenic line was obtained by examining ^13^C enrichment in Tre6P and intermediates of sucrose synthesis. During photosynthesis in ^13^CO_2_, enrichment rises rapidly to 85%–90% in Calvin–Benson cycle intermediates and to slightly lower levels in intermediates of sucrose synthesis like glucose 6-phosphate (Glc6P) and UDP-glucose (UDPGlc; Szecowka et al., 2013). Enrichment in Tre6P, Glc6P, and UDPGlc was high (74%–80%) at the end of the ZT2–ZT8 pulse in both *TPS29.2* and *AlcR* (Figure 7B) as expected if these metabolites are largely synthesized from newly assimilated C. Enrichment in these metabolites was much lower at the end of the ZT14–ZT20 pulse in *AlcR* (48%–53%), as expected if the C in these metabolites were derived in part from degradation of preformed (and hence, unlabeled) starch (for detailed studies of the reasons for incomplete enrichment of these intermediates, see Xu et al., 2021; Wieloch et al., 2022). This decline in enrichment between the early and the late pulse was much less in *TPS29.2*, consistent with the idea that elevation of Tre6P restricted starch degradation in *TPS29.2* relative to *AlcR*. Taken together, these changes in enrichment indicate that the rate of starch degradation between ZT14 and ZT20 in *AlcR* and *TPS29.2* is about 60% and 20%, respectively, of the rate of C fixation.

It was recently reported that long-term induction of the AlcR protein can lead to growth defects in Arabidopsis (Randall, 2021). It is very unlikely that the outcomes of our experiments were affected by this problem. Our experimental design involved very short-term induction by ethanol, and a comparison of control plants in which the AlcR protein was induced on its own with plants in which AlcR induced a *TPS* gene. Further, the responses of starch and other metabolites in the control induced *AlcR* plants (Figure 6, B–F) were essentially the same as in wild-type plants in similar conditions (Figures 1–3; Figueroa et al., 2016; Fünfgeld et al., 2021; see also below).

### Modification of the arithmetic division equation to include inhibition of starch degradation by Tre6P

We modified the skeletal arithmetic division equation (Model 1) to include a term that links the capacity for starch degradation to the rate of photosynthesis, such that starch degradation is inhibited during photosynthesis but is progressively activated as photosynthesis rate (*A*) falls, that is (*R*_d_* = (*S/T*)(1-A), Supplemental Calculation S2, Model 2). Based on the results in Figures 6 and 7; Supplemental Figure S5, we nominally ascribed this inhibition to Tre6P. We parameterized the model such that a 50% decrease in the rate of photosynthesis led, via a decline in sucrose and hence Tre6P levels, to a two-fold stimulation of starch degradation (see above, Figure 7B; Supplemental Figure S5). This modification of the equation reduced the discrepancy between the simulated and measured rates of starch mobilization during a subjective night. In particular, the modified equation correctly predicted that the rate of starch accumulation at high light would fall to a low positive value whereas the fall at low light would result in net starch loss (Figure 4C, right-hand display). This is because Tre6P is higher and therefore restricts starch degradation more strongly in high light than in low light. Like the unmodified equation, the modified equation correctly predicted the time-of-day dependent response to a decrease in light intensity (Figure 4D, right-hand display).

### Starch degradation maintains C availability as photosynthesis declines toward dusk in long-day conditions

Fernandez et al. (2017) proposed that a rising rate of starch degradation before dusk might buffer C availability and growth against the gradual decrease in the rate of photosynthesis at twilight, when irradiance and the rate of photosynthesis falls in natural light regimes. This idea was supported by the observation that sucrose levels are maintained at dusk in sinusoidal light regimes and in plants grown in natural light regimes (Annunziata et al., 2017). However, the most direct way to test this idea would be to investigate the rate of growth at twilight. In principle, buffering might be expected to occur in both short and long photoperiods. We explored the idea first using plants grown in long days, since starch degradation clearly increases in the light in the latter part of these long photoperiods.

We first used a whole plant C balance model (Sulpice et al., 2014) to simulate how C availability responds to a gradual decrease in photosynthesis at dusk (Supplemental Figure S7; Supplemental Calculation S3). We assumed that starch was not fully exhausted at dawn, as is indeed often the case in long-day conditions (see “Introduction,” also Figure 4E and below for more data). The model was applied to plants growing in an 18-h photoperiod, assuming that 33% of fixed C is allocated to starch (see Sulpice et al., 2014; Mengin et al., 2017) and that this value is not strongly affected by light intensity (see Mengin et al., 2017). When the model parameters were specified such that starch degradation occurred only in the dark, there was a transient drop in C availability during both the dawn and the dusk twilight (Supplemental Calculation S3, Model 0). This simulation also predicted that C availability in an 18-h photoperiod would be higher at night than in the daytime. When parameters were specified such that starch was degraded at a rate set by the skeletal arithmetic division equation, the transient trough in C availability during the dawn and dusk twilight was abolished, indeed, there was a transient overshoot (Supplemental Calculation S3, Model 1). When the skeletal equation was modified to include a photosynthesis-dependent restriction of starch degradation, predicted C availability changed smoothly during twilight (Supplemental Calculation S3, Model 2).

**Figure 8:**
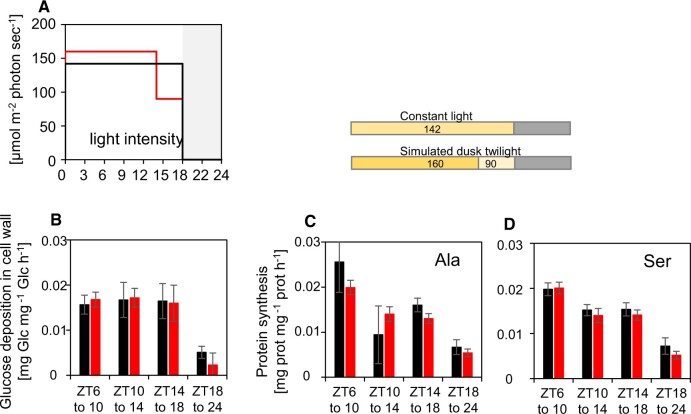
Rates of cell wall synthesis and protein synthesis in a simulated dusk twilight in plants growing in long-day conditions, measured by ^13^CO_2_ labeling. Plants were grown in an 18-h light/6-h dark cycle with a light intensity of 142 µmol m^−2^ s^−1^ throughout the light period or with a light intensity of 160 µmol m^−2^ s^−1^ from ZT0 to ZT14 and a light intensity of 90 µmol m^−2^ s^−1^ from ZT14 to ZT18 (simulated dusk twilight) (A). Rates (fractional increase per day) of cell wall synthesis (B) or protein synthesis estimated from ^13^C labeling in Ala (C) or Ser (D). Rates of protein synthesis were estimated as the increase in enrichment in Ala or Ser in protein in a given time interval, corrected for incomplete enrichment of free Ala or Ser (see Ishihara et al., 2015, 2017). The estimated synthesis rates are shown for constant light during the light period and simulated dusk twilight. Error bars for the rates of glucose incorporation in the cell wall and protein synthesis were calculated using the standard error of the mean for Gaussian error propagation. Time resolved data for starch, sucrose and maltose levels, ^13^C enrichment in free Ala and Ser, and ^13^C incorporation into glucose in the cell wall fraction and Ala and Ser in the protein fraction are provided in Supplemental Figure S8. Note that precursor pools are either labeled directly from fixed ^13^CO_2_ or, if starch or other reserves are being remobilized, from ^13^C incorporated into these reserves earlier in the 24-h cycle. Underlying data are provided in Supplemental Data Set 10. A similar experiment is shown in Supplemental Figure S9.

We next used an experimental approach developed in Ishihara et al. (2015, 2017) to test the prediction that starch degradation buffers growth against a decrease in photosynthesis at the dusk twilight (Figure 8; Supplemental Figure S8). In this method, plants are supplied continuously from dawn onwards with ^13^CO_2_. As starch levels are relatively low at dawn, the ^13^C enrichment in starch is similar to that in the supplied ^13^CO_2_. Growth rates are determined as: (1) the rate of accumulation of ^13^C in glucose in the cell wall fraction, representing synthesis of cellulose and the backbone of hemicellulose and (2) the rate of accumulation of ^13^C in protein. Most free amino acids show slow and incomplete labeling, which complicates or prevents their use for estimation of protein synthesis rates (see Ishihara et al., 2015 for details). Free Ala and Ser show a rapid rise to high enrichment and retain high enrichment during the night, so we monitored incorporation of ^13^C into these amino acids in protein, and corrected these values for the incomplete enrichment in free Ala and Ser to calculate the rate of protein synthesis (Ishihara et al., 2015).

Plants were grown in an 18-h photoperiod in a conventional step (light-on, light-off) light regime at 142 µmol m^−2^ s^−1^, or in a simulated twilight regime with 14 h at 160 µmol m^−2^ s^−1^ followed by 4 h at 90 µmol m^−2^ s^−1^ (Supplemental Figure 8A; Supplemental Data Set 11). This decrease in irradiance at twilight approximately halved the rate of photosynthesis (see also above). The total photon flux per 24-h cycle (daily light integral; DLI) was similar in the two regimes. A switch to a sustained low light intensity was used rather than a sinusoidal decline in light intensity because this provided a simpler experimental system in which to analyze labeling patterns and flux. On the harvest day, plants supplied continuously with ^13^CO_2_ from dawn were harvested at ZT6, ZT10, ZT14, ZT18, and ZT24. They were analyzed for starch, sucrose, and reducing sugars, and by gas chromatography-mass spectrometry (GC–MS) to determine ^13^C enrichment in glucose in the cell wall polysaccharides, in free Ala and Ser, and in Ala and Ser in protein.

As already seen, starch accumulation continued until dusk in the step light regime, although with falling rates, whereas starch content declined between ZT14 and ZT18 in simulated twilight (Supplemental Figure 8B). Sucrose levels were similar or higher in simulated twilight than the step light regime (Supplemental Figure 8C). Maltose levels started to rise perceptibly between ZT8 and ZT12 in the step regime and rose more strongly in simulated twilight (Supplemental Figure 8D). Enrichment in free Ala and free Ser was high (>75%) except for Ser at the end of the night (Supplemental Figure 8, E–F). ^13^C enrichment in cell wall glucose (Supplemental Figure 8G) and in Ala and Ser in protein (Supplemental Figure 8, H–I) rose steadily during the light period and more slowly at night, as seen previously (Ishihara et al., 2015). Crucially, rates of enrichment in cell wall and protein in the last 4 h of the light period were the same, irrespective of whether plants were in constant light or subjected to simulated twilight (Figure 8, A–C). A similar result was obtained in a separate experiment (Supplemental Figure S9). These experiments show that cell wall and protein synthesis are unaffected by a drop in light level at the end of the day. The data are consistent with increased provision of C from starch degradation at the end of the light period so that growth is maintained as the contribution from photosynthesis declines.

### Starch degradation maintains C availability as photosynthesis declines toward dusk in short days

We next asked whether starch degradation can also buffer growth in the evening twilight in short-day conditions. In short days, dusk occurs at a time in the 24-h cycle when there is little starch degradation in the light (see Figures 1–3; Fernandez et al., 2017), and decreases in light intensity before dusk in short-day plants do not reduce starch accumulation by much more than expected from the reduction in the rate of photosynthesis (see Figures 2, A and 3, A). Nevertheless, three considerations led us to suspect that even low rates of starch degradation might buffer C availability during the dusk twilight in short-day conditions. The first was that a larger proportion of the fixed C accumulates as starch in short photoperiods and daytime growth is slower than in long photoperiods. In short photoperiods starch accounts for up to 65% of the fixed C (Mengin et al., 2017), thus, the rate of daytime growth is lower and even a small decrease in net starch accumulation might lead to a substantial increase in the availability of C for growth. The second was that larger allocation to starch will lead to starch content rising more quickly in short-day than in long-day plants. Assuming that the rate of degradation, *R*_d,_ rises with starch content, S, this will result in higher rates of starch degradation early in the light period in short-day plants than in long-day plants. The third was that the rate of starch degradation at night is much lower in plants growing in a short photoperiod than in a long photoperiod (see e.g. Sulpice et al., 2014; Mengin et al., 2017), thus even low rates of degradation during the dusk twilight might suffice to prevent a transient drop in growth.

We first explored these ideas by modeling the impact of a light drop on photosynthesis, starch synthesis, starch degradation, and the availability of C for growth in a 6-h photoperiod (Supplemental Figure S10; Supplemental Calculation S4), using a similar approach to that described above for long days (Supplemental Figure S7) except that we parameterized the model such that 65% of fixed C is allocated to starch, based on empirical data for Arabidopsis in short-day conditions (Sulpice et al., 2014; Mengin et al., 2017). Dusk twilight was simulated using decreases in light intensity at ZT4 that led to a 50% or 75% decrease in the rate of photosynthesis. The model predicted that in the absence of starch degradation these treatments would lead to a strong fall in the availability of C for growth. Permitting starch degradation in the light almost completely reversed the fall after a 50% decrease in photosynthesis, and partly reversed the effect of a 75% decrease in photosynthesis (Supplemental Figure 10A). These simulations predict that starch degradation could indeed buffer growth during the evening twilight in short-day plants. The high allocation of fixed C into starch in short days is essential for the buffering mechanism. In a simulation in which only 35% of fixed C was accumulated as starch in short days the predicted rate of starch degradation between ZT4 and ZT6 did little to buffer growth in the dusk twilight (Supplemental Figure 10B).

**Figure 9:**
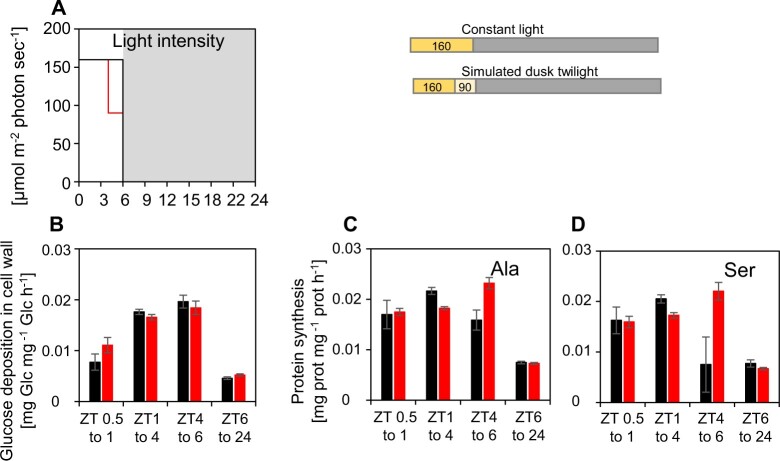
Rates of cell wall and protein synthesis in a simulated evening twilight in short-day conditions, measured by ^13^CO_2_ labeling. Plants were grown in a 6-h light/18-h dark cycle at a light intensity of 160 µmol m^−2^ s^−1^ throughout the light period (step light regime) or with a light intensity of 160 µmol m^−2^ s^−1^ from ZT0 to ZT4 and a light intensity of 90 µmol m^−2^ s^−1^ from ZT4 to ZT6 (simulated dusk twilight) (A). Rates (fractional increase per day) of cell wall synthesis (B) or protein synthesis, estimated from ^13^C labeling in Ala (C) and Ser (D). The rates of protein synthesis were estimated as the increase in enrichment in Ala or Ser in protein in a given time interval, corrected for incomplete enrichment of Ala or Ser in the free amino acid pool (see Ishihara et al., 2015, 2017). The estimated synthesis rates are shown for constant light during the light period and simulated dusk twilight. Error bars for the rates of glucose incorporation in the cell wall and protein synthesis were calculated using the standard error of the mean for Gaussian error propagation. Time resolved data for starch, sucrose, and maltose levels, ^13^C enrichment in free Ala and Ser, and ^13^C incorporation into glucose in the cell wall fraction and Ala and Ser in the protein fraction are provided in Supplemental Figure S11. Note that precursor pools are either labeled directly from fixed ^13^CO_2_ or, if starch or other reserves are being remobilized, from ^13^C incorporated into these reserves earlier in the 24-h cycle. Underlying data are provided in Supplemental Data Set 12.

These ideas were experimentally tested by performing ^13^CO_2_ labeling experiments. Plants were grown in a 6-h photoperiod with a simulated twilight (4 h of 160 µmol m^−2^ s^−1^ followed by 2 h at 90 µmol m^−2^ s^−1^) or, as a control, in a 6-h photoperiod at 160 µmol m^−2^ s^−1^ (Figure 9A; Supplemental Figure S11A; Supplemental Data Set 12). Irradiance was not adjusted to provide the same DLI in both treatments. Plants supplied with ^13^CO_2_ from dawn were harvested at ZT0, ZT4, ZT6, and ZT24. After the decrease in light intensity at ZT4, starch accumulation was slowed slightly (Supplemental Figure S11B), sucrose remained high (Supplemental Figure S11C) and, as observed in Figure 2, there was a small increase in maltose (Supplemental Figure S11D). ^13^C incorporation into free Ala and Ser (Supplemental Figure S11, E–F), glucose in the cell wall (Supplemental Figure S11G), and Ala and Ser in protein (Supplemental Figure S11, H–I) revealed that cell wall and protein synthesis were three- to four-fold slower at night than in the daytime in these short-day conditions. Crucially, cell wall synthesis was maintained at the same rate in the simulated twilight as in high light (Figure 9B) and protein synthesis might even have increased in the simulated twilight (Figure 9, C and D). Thus, as in long days, starch degradation at the end of the day in short days provides C to permit maintenance of growth as C from photosynthesis falls.

### Starch degradation continues in the first part of the light period in long-day conditions

We next investigated the contribution of starch degradation to events after illumination at dawn. Plants growing in long days at moderate or high irradiance contain substantial amounts of starch at dawn and, after reillumination, there is typically a 2- to 3-h delay before net starch accumulation starts (Hädrich et al., 2012; Sulpice et al., 2014; Figueroa et al., 2016; Fünfgeld et al., 2021). This lag is unlikely to be due to a slow onset of starch synthesis, because ADPGlc levels rise rapidly after dawn (Figueroa et al., 2016; Fünfgeld et al., 2021). We carried out two experiments to discover whether the lag is due to continued degradation of starch in the first part of the light period.

**Figure 10:**
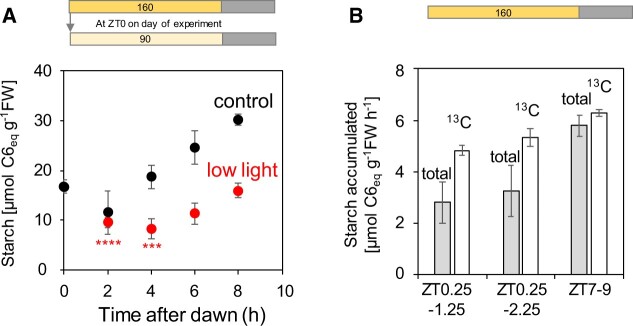
Starch degradation in the first hours of the light period in long-day conditions. A, Plants were grown in a 18-h light/6-dark cycle at 160 µmol m^−2^ s^−1^ for 21 days and then either left at growth irradiance (black symbols) or transferred to 90 µmol m^−2^ s^−1^ (red symbols). Plants were harvested just before dawn and at 2-h intervals for the first part of the light period. The results are given as mean and 95% confidence limits (*n* = 5 except ZT8 where *n* = 4, each replicate contained five rosettes). Significant differences compared to the ZT0 value were identified by two-tailed *t* test with equal variance (*P < 0.05, **P < 0.01, ***P < 0.001, ****P < 0.0001). The underlying data are provided in Supplemental Data Set 13. B, Plants were grown for 21 days in an 18-h light/6-h dark cycle at 160 µmol m^−2^ s^−1^ and on the 22nd day were supplied with ^13^CO_2_ between ZT0.25 and ZT1.25, or between ZT0.25 and ZT2.25 or between ZT7 and ZT9. Plants were harvested at the start and end of each labeling interval. The display shows the estimated rate of accumulation of total starch (gray bars) and ^13^C starch (white bars). The contents of ^12^C and ^13^C starch, which were used to calculate these rates, are provided in Supplemental Figure S12 and Supplemental Data Set 14. Error bars were calculated using the se of the mean (*n* = 3–4) for Gaussian error propagation. Comparable experiments with short-day-grown plants are provided in Supplemental Figure S11 and Supplemental Data Sets 15 and 16.

In the first experiment, plants were grown in 18-h photoperiods at 160 µmol m^−2^ s^−1^ and either left at this light intensity (control plants) or transferred at dawn to 90 µmol m^−2^ s^−1^ (Figure 10A; Supplemental Data Set 13). In control plants, changes in starch levels up to ZT2 were not statistically significant, after which levels started to rise as seen in previous studies. In plants transferred to a lower light intensity at dawn, there were statistically significant falls in starch levels at ZT2 or ZT4, after which levels started to rise. This decrease in starch content demonstrates that starch degradation can continue in the first part of the light period. In the second experiment, plants were grown in an 18-h photoperiod at 160 µmol m^−2^ s^−1^ and supplied with ^13^CO_2_ between ZT0.25 and ZT1.25, between ZT0.25 and ZT2.25 and, as a control treatment, between ZT7 and ZT9 (Figure 10B; Supplemental Data Set 14). As previously seen, during the ZT7 to ZT9 pulse the rate of ^13^C-starch accumulation resembled the rate of total starch accumulation and there was no loss of ^12^C-starch. These data are consistent with the absence of starch degradation during this period. In contrast, during pulses early in the light period, ^13^C-starch accumulated twice as fast as total starch and ^12^C-starch showed a significant decline. A replicate experiment gave a similar result (Supplemental Figure S12) Together, these results show that starch degradation can continue for up to 4 h into the light period when plants are growing in long-day conditions. Possible mechanisms underlying this phenomenon are proposed in the “Discussion.”

### There is no starch degradation in the early part of the light period in short-day conditions

We also investigated if starch degradation continues in the early part of the light period in short day-grown plants. In a first experiment, we provided a pulse of ^13^CO_2_ in the first 2.25 h after dawn (Supplemental Figure S13; Supplemental Data Set 15). After illumination, total starch accumulated linearly with time (Supplemental Figure S13A), as typically seen in short-day plants (Gibon et al., 2009; Sulpice et al., 2014; Mengin et al., 2017). Crucially, the rate of ^13^C-starch accumulation resembled the rate of total starch accumulation and there was no loss of ^12^C-starch (Supplemental Figure S13, B and C). Wild-type plants have very low levels of starch at dawn in short photoperiods. We, therefore, repeated these experiments using a mutant lacking *DISPROPORTIONATING ENZYME 1*, which has similar or higher starch levels at dawn in short photoperiods to those found in wild-type plants in long photoperiods, but is still able to degrade starch at rates close to those in the wild-type (Critchley et al., 2001). The *dpe1* mutant was grown in a 6-h photoperiod and pulsed with ^13^CO_2_ between ZT0.25 and 2.25. After illumination, total starch accumulated linearly with time (Supplemental Figure S13D and Supplemental Data Set 16), the rate of ^13^C-starch accumulation resembled the rate of total starch accumulation and there was no loss of ^12^C-starch (Supplemental Figure S13, E and F). These results showed that there is little or no degradation of starch at the start of the light period in short-day plants, even if they still contain substantial amounts of starch at dawn.

### Starch degradation slows down after subjective dawn in extended continuous light

In addition to investigating whether starch degradation continues for some time after dawn in a light-dark cycle, we investigated the response after subjective dawn in continuous light conditions. Although starch content plateaued or even declined from about ZT14 onwards it did not show a clear increase after subjective dawn (see e.g. Figure 4A). Assuming constant rates of starch synthesis, the skeletal equation *R*_d_ = *S/T* would predict an increase in starch content after subjective dawn because time to the next anticipated dawn (i.e. T) suddenly increases. This apparent discrepancy was investigated in material from an experiment in Fernandez et al. (2017) in which plants growing in a 12-h light/12-h dark cycle at 160 µmol m^−2^ s^−1^ were transferred to continuous low light (90 µmol m^−2^ s^−1^) and sampled until ZT33 (9 h after the first subjective dawn). As reported in Fernandez et al. (2017), *A* and ADPGlc content remained high throughout the continuous light treatment, starch content plateaued by ZT15, showed a slight decline between ZT15–ZT24 followed by a slight rise between ZT24–ZT33 (the slopes in these time intervals were marginally different, *P* = 0.051), and sucrose, glucose, and Glc6P levels rose in the subjective night and declined after ZT24 (Supplemental Figure S14). These responses are consistent with a lower rate of starch degradation after subjective dawn. As in many other experiments, maltose content rose steeply from ZT12 onwards to reach high levels at the end of the subjective night. After the subjective dawn, maltose content declined by about 2-fold at ZT27 and almost 10-fold at ZT33. The levels at ZT27 and ZT33 were much higher than at ZT3 and ZT9 (Supplemental Figure S14). These maltose measurements provide qualitative evidence that in continuous light conditions starch degradation is gradually inhibited after subjective dawn, although rates may remain higher than at a corresponding time in a light dark cycle. This may explain why starch content does not rise strongly after ZT24. It might be noted that starch content was much higher after subjective dawn in continuous light than after dawn in a light/dark cycle. Accordingly, the skeletal arithmetic division equation would predict a high degradation rate after subjective dawn than after dawn in a light/dark cycle.

## Discussion

We have investigated whether the regulation of starch degradation in the light may be similar to regulation of starch degradation in the dark. We have also investigated whether starch degradation in the light contributes to maintenance of C availability during the dusk twilight and optimization of C allocation to starch in different photoperiods

### Starch degradation in the light increases with time after dawn in a manner largely predicted by the skeletal arithmetic division equation

In the dark, the circadian clock paces degradation such that starch is almost but not completely exhausted at dawn (Graf et al., 2010; Scialdone et al., 2013; Flis et al., 2019; Smith and Zeeman, 2020). Fernandez et al. (2017) reported that the rate of degradation in the light speeds up with time after dawn, leading by about ZT14 to a slowing down or reversal of net starch accumulation. The timing of this effect was advanced in the short period *lhy cca1* clock mutant and delayed in the long period *prr7 prr9* clock mutant. These observations were consistent with the idea that the clock also regulates starch degradation in the light.

The Arabidopsis clock is largely dawn dominant (Edwards et al., 2010; Flis et al., 2015, 2016). If the circadian clock regulates starch degradation in the light, the time at which degradation speeds up should therefore depend on the timing of dawn rather than dusk. Comparison of Arabidopsis growing in a 6-h and an 18-h photoperiod provided three lines of evidence that temporal kinetics of degradation indeed depend on the time that has elapsed since dawn, and are largely independent of the timing of dusk. First, irrespective of whether plants were grown in a short photoperiod or a long photoperiod before transferring them into continuous light, net starch accumulation plateaued by 14–16 h after dawn (Figure 1, A–B; Supplemental Figure S1A). Second, when plants were grown in a 6-h photoperiod, a decrease in photosynthesis due to a decrease in light intensity 2 h before dusk led to a commensurate slowing of starch accumulation (Figure 2A). In contrast, when plants were grown in an 18-h photoperiod, a decrease in photosynthesis due to an identical decrease in light intensity 3 h before dusk led to net loss of starch (Figure 2B). Third, ^13^CO_2_ labeling studies revealed that starch degradation was slow in the last 2 h of a 6-h photoperiod in short day-grown plants, and was slow in the middle of the light period but fast in the last 4 h of the light period in long day-grown plants (Figure 3). These results provide strong evidence that the propensity for starch degradation rises with time after dawn, and that this response is largely independent of the timing of dusk. Our measurements of maltose content, a qualitative proxy for starch degradation, provided independent evidence that the rate of starch degradation increases with time after dawn, and also indicated that the rate depends on the current starch level (Figures 1, C, D, 2, C and D; Supplemental Figure S1). Our results do not exclude a slight dependence on photoperiod duration. This might even be expected because, in Arabidopsis, clock phase is delayed by 2–3 h relative to dawn in an 18-h compared to a 6-h photoperiod (see below for more discussion).

These observations led us to ask whether the pattern of starch degradation in the light could be explained by concepts that were developed to explain starch degradation during the night. Various models have been proposed to explain the pattern of starch degradation in leaves in the night, including the sugar homeostasis models (Seki et al., 2017; Webb et al., 2019) and the arithmetic division model (Scialdone et al., 2013). The former indeed predicted that a higher rate of starch degradation late in the light period would be required to maintain sugar homeostasis in long days. For quantitative analysis of our data, however, we chose to use the skeletal equation of the arithmetic division model. This equation allowed us to quantitatively predict starch degradation rate and starch content, based on explicit variables that were measured in our study. According to the skeletal equation of the arithmetic division model (Scialdone et al., 2013), the rate of starch degradation, *R*_d_, = *S/T* where *S* is the amount of starch and *T* is time to dawn. This model predicts the observed kinetics of starch degradation in the dark in a wide range of conditions (see “Introduction”). We used this equation to simulate the kinetics of starch accumulation after transfer to continuous light (Figure 4B). Our simulation assumed that the rate of starch synthesis is constant with time; evidence for this assumption is provided by the near-constant rate of photosynthesis (Supplemental Figure S4; Fernandez et al., 2017) and level of ADPGlc in continuous light (Figure 6E, see also Figueroa et al., 2016; Fünfgeld et al., 2021). ADPGlc is the dedicated precursor for starch synthesis and the product of ADP-glucose pyrophosphorylase, which exercises strong control over the rate of starch synthesis (Ballicora et al., 2004; Stitt et al., 2010; Hädrich et al., 2011). The skeletal arithmetic division equation predicted a progressive rise in the rate of degradation with time after dawn, as starch content rises and time to the next dawn decreases (Figure 4B). It predicted that the response to a decrease in light intensity would be a slowing of starch accumulation when the decrease is imposed early in the 24-h cycle, and net starch degradation when the decrease is imposed later in the 24-h cycle (Figure 4D). It also predicted that a decrease imposed early in the 24-h cycle would lead to a net decline in starch if plants enter the light period with a high starch content (Figure 4F). However, although the skeletal equation successfully simulated the observed changes in starch content in most circumstances, this was not universally the case. In particular, in continuous high irradiance, the equation predicted rapid net loss of starch by ZT18, whereas the observed response was usually a slowing down of accumulation or plateauing of the starch content (compare Figure 4, A and B, left hand display). This discrepancy indicated that some further factor restricts starch degradation in the light, especially in high light.

### The sucrose signal Tre6P can restrict starch degradation in the light

We considered whether the rate of starch degradation in the light might be restricted by gated responses to light levels. However, a decrease in CO_2_ concentration affected starch accumulation in a manner similar to a decrease in light intensity (Figure 5). This led us to suspect that starch degradation may be restricted by changes in metabolism that occur as a result of rapid C fixation.

Increasing rates of photosynthesis typically lead to an increase in sucrose content (Servaites et al., 1989; Annunziata et al., 2017; Mengin et al., 2017) and an increase in the level of Tre6P, which signals sucrose availability (Supplemental Figure S5, see also Annunziata et al., 2017). Indeed, Tre6P levels correlate with sucrose content across many conditions (Lunn et al., 2006, 2014; Yadav et al., 2014; Figueroa and Lunn, 2016; dos Anjos et al., 2018; Fichtner et al., 2020). We earlier used transgenic lines in which Tre6P levels can be inducibly elevated to show that Tre6P inhibits starch degradation in the dark (Martins et al., 2013; dos Anjos et al., 2018). We used the same lines to investigate whether Tre6P restricts starch degradation in the light (Figures 6 and 7), by inducing a rise in Tre6P at a time at which starch degradation in the light was rising in control plants. In the induced plants a circa 2.5-fold increase in Tre6P reduced the rate of starch degradation and reduced maltose levels relative to those in control plants, and starch continued to accumulate. The level of ADPGlc was unaltered indicating that starch synthesis was unaffected (see also Figueroa et al., 2016). Whilst it is challenging to quantify precisely the rate of starch degradation in the light, analysis of ^13^C-labeling patterns indicated that a change in Tre6P leads to a roughly inversely proportional change in the rate of starch degradation (Figure 7B). This resembles the relationship between Tre6P levels and the rate of starch degradation in the dark (Martins et al., 2013; dos Anjos et al., 2018).

Whilst our results show that Tre6P plays a major role, they do not exclude the possibility that further factors also exert feedback regulation on starch degradation in the light. For example, changes in light intensity might potentially affect starch metabolism via redox signaling (Glaring et al., 2012; Thormählen et al., 2013; Krasensky et al., 2014), or changes in energy supply or levels of phosphorylated intermediates (Stitt et al., 1982, 1983; Badger et al., 1984; Dietz and Heber, 1984; Stitt et al., 2010; Borghi et al., 2019). However, we suspect that any such effects are minor, because starch accumulation responds in a qualitatively similar manner to a decrease in light intensity and a decrease in CO_2_ concentration that have opposing effects on energy supply (Figure 5). Another potential complication is that starch is sometimes degraded in the light via different pathways to those that operate in the dark. At night BAM3 is the major amylolytic activity (Fulton et al., 2008; Kötting et al., 2010; Stitt and Zeeman, 2012). Under drought stress in the light, starch is degraded via the amylolytic enzymes AMY3 and BAM1 (Valerio et al., 2010; Seung et al., 2013; Thalmann et al., 2016; Zanella et al., 2016). This pathway is enhanced by ABA-dependent transcriptional activation of *BAM1* and *AMY3*. It is unlikely to be involved in the stimulation of starch degradation when irradiance is decreased because a decrease in irradiance will lead to a decrease in stomatal aperture (Kölling et al., 2015) and an increase rather than a decrease in leaf water potential. It is also unlikely to explain the gradual increase in the rate of starch degradation with time in the light in well-watered plants. When photorespiration is rapid, starch may be degraded by phosphorylase to maintain levels of Calvin–Benson cycle intermediates (Weise et al., 2006, 2011; reviewed in Stitt et al., 2021). However, this is unlikely to contribute to the stimulation of starch degradation in response to moderate decreases in irradiance, as these lead to only small changes in the levels of phosphorylated intermediates (Stitt et al., 1983, 2021; Badger et al., 1984; Dietz and Heber, 1984; Borghi et al., 2019).

At night, Tre6P acts as a feedback inhibitor of starch degradation (Martins et al., 2013), lowering the rate below that required to exhaust starch at dawn (dos Anjos et al., 2018). Tre6P likely exerts an analogous influence in the light. A modified form of the skeletal arithmetic division equation, *R*_d_* =  (*S/T*)(1-A) that included a term for feedback inhibition by Tre6P simulated the response of starch accumulation after transfer to continuous high or low irradiance (compare Figure 4, A and B, right-hand display). In both the dark (Martins et al., 2013) and the light (Figure 6), starch degradation is rapidly inhibited after an induced increase of Tre6P, indicating Tre6P acts rather directly to slow down starch degradation. In both cases, there is a marked decrease in maltose, revealing that Tre6P inhibits an early step upstream of maltose formation in the pathway of starch degradation. The precise target and mechanism are unknown (Martins et al., 2013). Formally, the inhibitory action of Tre6P may be analogous to the response in several mutants with partial lesions in the starch degradation pathway. Many of these mutants show incomplete mobilization of starch at night, with the proportion of the dusk content that remains at dawn being fixed for a given mutant (Scialdone et al., 2013). Indeed, this response was seen in the dark in the *sweet11;sweet12* mutant, which has impaired sucrose export and elevated levels of both sucrose and Tre6P (dos Anjos et al., 2018). It might be noted that the modified equation will simplify to *R* = *S/T* in wild-type plants in the dark in conditions where sucrose and Tre6P are low.

### Starch degradation continues after dawn in long photoperiod-grown plants

When plants are grown in long photoperiods, there is typically a two- to 3-h delay after illumination until rapid starch accumulation starts (Sulpice et al., 2014; Figueroa et al., 2016; Fünfgeld et al., 2021, see also Figure 10A). Whilst there may be a short delay until full rates of photosynthesis are attained and starch synthesis starts, this cannot explain the long delay before starch accumulation starts because high levels of ADPGlc are established soon after illumination (Figueroa et al., 2016; Fünfgeld et al., 2021). Our results show that the major reason for the delay is that starch degradation continues for at least 2 h after dawn in long photoperiod-grown plants with high starch contents at dawn (Figure 10, A and B; Supplemental Figure S12).

Continuation of starch degradation after dawn is at first sight inconsistent with the arithmetic division model, in which T is proposed to approach zero at dawn. A possible explanation is that after dawn there may be a delay until T is reset. Analysis of maltose and other metabolite levels in long photoperiod-grown plants transferred to continuous light showed that starch degradation can continue for some hours after subjective dawn, although at a falling rate (Supplemental Figure S14). Furthermore, there is a 2- to 3-h delay in clock phase in long photoperiods compared to short photoperiods (see Fowler et al., 1999; Matsushika et al., 2000; Edwards et al., 2010; Flis et al., 2015, 2016). This leads to the dawn *LHY* and *CCA1* transcripts peaking 1 to 2 h after dawn, and a corresponding delay in the timing of clock outputs (Millar and Kay, 1996; Seaton et al., 2015; Flis et al., 2016). It is possible that the dynamics of T are similarly delayed in long-day conditions. The observation that there is no detectable starch degradation after dawn in a 6-h photoperiod (Supplemental Figure S13) is consistent with this explanation.

Alternatively, viewed in terms of the sugar homeostasis models, it is possible that the presence of substantial amounts of starch and sugars at dawn in long-day plants modifies sugar inputs to the core circadian clock (Haydon et al., 2013; Seki et al., 2017; Frank et al., 2018; Webb et al., 2019) and associated pathways (e.g. including bZIP63; Viana et al., 2021) that are proposed to regulate starch degradation, and that this leads to continued degradation of starch for the first hours of the light period. Using a model that explores the consequences of a pattern of starch degradation that is optimized to maintain constant levels of sucrose, Seki et al. (2017) predicted high rates of starch degradation for a time after dawn in long-day conditions, as is seen in our experimental data. Whilst this might be the consequence of a mechanism like that envisaged in sugar homeostasis models, it could also be generated by the arithmetic division model in combination with the observed shift in clock phase in long days.

### Starch degradation will buffer growth and signaling against falling rates of photosynthesis at twilight in natural light regimes

In the field, irradiance increases gradually in the morning, and decreases gradually in the evening. It is a reasonable assumption that the complex networks that regulate metabolism and growth have evolved to optimize growth in such conditions (Annunziata et al, 2017, 2018), rather than in response to the sudden switches between light and darkness that are typically used in experiments in controlled conditions. These considerations led us earlier to propose that starch degradation might buffer C availability and growth against a falling rate of photosynthesis as the light intensity declines during the twilight (Servaites et al., 1989; Fernandez et al., 2017, see also Fondy et al., 1989).

We used a whole plant C balance model to explore how starch degradation might modify the response of growth to low light intensities during the morning and evening twilight in long day-grown plants (Supplemental Figure S7). The model predicted that if there is no starch degradation in the light, there would be large transient inhibitions of growth during the morning and evening twilight (Model 0), and that starch degradation in the light would buffer against these transient inhibitions (Model 1). In the dusk twilight, for example, the falling supply of C from photosynthesis as irradiance declines is buffered by a rising supply of C from starch degradation. The smoothest transition was obtained using a modified model (Model 2) that included inhibition of starch degradation by Tre6P. Using ^13^CO_2_ labeling, we experimentally confirmed that cell wall and protein synthesis is maintained in the face of a simulated dusk twilight (Figure 8; Supplemental Figures S8 and S9). It is likely that starch degradation plays an analogous role during the dawn twilight, although this prediction was not experimentally validated.

A buffering effect of starch degradation on growth during the evening twilight was also predicted (Supplemental Figure S10) and observed (Figure 9; Supplemental Figure S11) in short day-grown plants. This occurs despite starch degradation being relatively slow at the point in the 24-h cycle at which dusk occurs in short day-grown plants. The ability of starch degradation to buffer growth during twilight in short-day plants reflects the higher proportion of the fixed C that is allocated to starch and the lower proportion that is allocated to growth in the light. As a result, starch content rises faster in short-day than in long-day plants, which promotes starch degradation earlier in the 24-h cycle, albeit at a slow rate. Since the growth rate in the light period is slow in short-day plants, even a low rate of starch degradation during twilight suffices to buffer growth against a decline in photosynthetically fixed C.

Starch degradation in the light may serve a further metabolic function in long-day conditions. In these conditions, there is a relatively low requirement for fixed C to be accumulated as starch to support growth and maintenance at night, because the night is short. Previous work has shown that the percentage of fixed C accumulated as starch during the day falls from 60% in a 6-h or 8-h photoperiod, to 33%–40% in a 12-h photoperiod, and to as little as 25% in an 18-h photoperiod (Sulpice et al., 2014; Mengin et al., 2017). The low value in an 18-h photoperiod was largely due to a delay before starch accumulation started, and a slowing down toward the end of the light period. The rate of accumulation in the middle of the light period resembled that in a 12-h photoperiod.

In addition, by maintaining sugar levels, starch degradation may reinforce signaling events during a natural twilight. Circadian-based measurement of photoperiod modulates many responses, including flowering via the CONSTANS/FLOWERING TIME pathway (Turck et al., 2008; Song et al., 2015). This pathway is positively modulated by Tre6P (Wahl et al., 2013) and might be promoted by maintaining high levels of sucrose and Tre6P during the dusk twilight. Furthermore, it was recently proposed that some environmental responses are controlled by a separate metabolism-based daylength measuring system (Liu et al., 2020; Gendron et al., 2021). By maintaining sugar levels high during a natural twilight, starch degradation will stabilize and even reinforce photoperiod responses that involve an interaction between photoperiod-sensing and sugar-signaling. More generally, starch degradation during twilight will buffer against a transient drop in sugar and energy levels and any resulting inhibition of growth and activation of C-starvation signaling as plants transition from the light to the dark in field conditions (Pal et al., 2013; Annunziata et al., 2017)

In conclusion, starch degradation can occur simultaneously with starch synthesis and has a major impact on the temporal kinetics of starch accumulation in the light. The pattern of starch degradation in the light can be predicted using concepts developed to explain the response of starch degradation in the dark, and can be simulated by a slightly modified version of the skeletal arithmetic division equation (Scialdone et al., 2013) in which degradation is additionally restricted by Tre6P (Martins et al., 2013; dos Anjos et al., 2018). Starch degradation in the light is of particular importance in buffering growth and signaling against low light during the twilight in natural light regimes. It can also help to minimize accumulation of excess starch in long photoperiods.

## Materials and methods

### Plant material

Arabidopsis (*Arabidopsis thaliana*) [L.] Heynh. Col-0 was grown on soil in controlled environment rooms as in Mengin et al. (2017) and Fernandez et al. (2017) in a 6-, 12-, or 18-h photoperiod (22°C, 160 µmol quanta m^−2^ s^−1^, 75% relative humidity). A list of all experiments with the experimental design, which data displays was generated from them and the location of the accompanying data sets is provided in Supplemental Table S1). Changes in the light regime before or on harvest day are described in the Figure legends and inserts in the Figure panels.

### Metabolite analysis

Rosettes were harvested under ambient light conditions and frozen immediately in liquid nitrogen. Sugars and starch were extracted and measured enzymatically (Stitt et al., 1989). Tre6P and ADPGlc were extracted and measured using high-performance anion exchange chromatography coupled to tandem mass spectrometry (Lunn et al., 2006, modified as in Figueroa et al., 2016). Maltose was extracted from 30-mg homogenized material with ice-cold 100% (v/v) methanol containing 1.31-mM ribitol as an internal standard, followed by phase separation using chloroform-water (Heise et al, 2014). The upper polar phase was used for methoxysilylation of metabolites with *N*-methyl-*N*-trimethylsilyltrifluoroacetamide followed by gas chromatography time-of-flight mass spectrometry (GC–TOF–MS) analysis using conditions and settings as in Lisec et al. (2006). Chromatography peaks were extracted and normalized using the ribitol peak area as in Szecowka et al. (2013). Maltose was quantified using a standard curve (three concentrations of maltose standard).

### Photosynthesis

Gas exchange was measured using a LI‐6400XT Portable Photosynthesis System fitted with the 6400‐17 Whole Plant Arabidopsis Chamber (Li‐Cor Biosciences GmbH, D‐61352 Bad Homburg, Germany; similar to Pyl et al., 2012; Sulpice et al., 2014). Plants were grown from seed in 6.5-cm-diameter pots specially designed to fit the measurement chamber. One plant was grown per pot. To avoid effects from soil respiration, the soil surface was covered with a black plastic film, and a slight overpressure was applied in the chamber, by restricting the exhaust air flow, to prevent movement of CO_2_ from the soil into the chamber air space.

### ^13^CO_2_ labeling

Starch synthesis and degradation were investigated using 2- to 6-h pulses (for details see figure legends and Supplemental Figure S1) of 99% ^13^CO_2_ as in Fernandez et al. (2017). The starch in the sample was enzymatically digested with α-amylase and amyloglucosidase. The glucose from the starch digestion was incubated with ATP and hexokinase to generate G6P that was analyzed by LC–MS/MS for estimation of ^13^C enrichment (Fernandez et al., 2017). Rates of cell wall and protein synthesis were investigated by providing ^13^CO_2_ continuously from dawn onwards, and plant material was analyzed and calculations performed precisely as in Ishihara et al. (2015, 2017). After methanol-chloroform-water phase extraction, polar phase was analyzed by GC–TOF–MS and the pellet was kept for protein and cell wall analysis (Ishihara et al., 2015). Protein was extracted from the pellet using 6-M urea/2M thiourea solution, hydrolyzed, and analyzed by GC–TOF–MS (Ishihara et al., 2015). The pellet residue after protein extraction was used for determination of ^13^C enrichment in cell wall. After removing starch (three sequential 16-h digestions with 30-U mL^−1^ α-amylase and 16.8-U mL^−1^ amyloglucosidase) and testing that no starch remained, the pellet was chemically digested and the released glucose determined by GC–TOF–MS as described (Ishihara et al., 2015, 2017).

### Imposition of a decrease in [CO_2_]

Plants were grown in a 12-h photoperiod at 150-μmol quanta m^−2^ s^−1^, 20°C for 24 days. At the start of the day of the experiment, plants were transferred to light of 105-μmol quanta m^−2^ s^−1^. For experiments in which [CO_2_] was decreased, individual plants were placed in a multi-chamber, computer-controlled, infrared gas analyzer-coupled system for the continuous measurement of gas exchange in whole-plant shoots or rosettes (ETH Gas Exchange System-2; Kölling et al., 2015; George et al., 2018), and rates of photosynthesis were continuously monitored. Control plants were placed beside the chambers, in the same environmental conditions. [CO_2_] inside the chambers was reduced from 380 to 195 ppm at ZT6 or ZT10, and plants from the chambers together with control plants that remained at 380 ppm CO_2_ were harvested 3 h after the [CO_2_] decreases were imposed (i.e. ZT10 and ZT13). The same procedure was followed for imposition of a decrease in light intensity at ZT6 and ZT10, from 105 to 65 μmol quanta m^−2^ s^−1^.

### Calculations

The change in starch content with time in the light was predicted as in the legend of Figure 4 and Supplemental Calculation S2. The predicted changes were compared with observed responses show in panels A, C, and E (see Fernandez et al., 2017; Supplemental Calculation S1 for a compilation of data for panel A; 2017) and Figure 2, A and C; Supplemental Figure S4, A and C for panel C; Supplemental Data Set S7 for panel E). Decreasing light intensity from 160 to 90 μmol quanta m^−2^ s^−1^ leads to a ∼50% inhibition of photosynthesis. The rate of starch synthesis, R_s_, was assumed to be constant with time in the light (as supported by the near constant levels of ADPGlc through an 18-h photoperiod (Figueroa et al., 2016; Fünfgeld et al., 2021), or after transfer to continuous light (Figure 6E). R_s_ was set as 1 relative units h^−1^ in growth irradiance and 0.5 relative units h^−1^ under lower irradiance that led an ∼50% inhibition of photosynthesis (the rate of starch synthesis is almost linear with irradiance (Mengin et al., 2017). Starch content at dawn was assumed to be negligible (see Figure 4A) in the simulations of Figure 4, B and D and about 33% of the value at ZT14 in the simulation of Figure 4F (similar to the relationship in the data sets that were simulated, Figure 4, A, C, and E, respectively). The rate of starch degradation was simulated using two different equations. In Equation (1), the rate of starch degradation, *R*_d_, = *S/T*, where *S* is the amount of starch and *T* is the remaining time to dawn. In Equation (2), the rate of starch degradation, *R*_d_*, =  (*S/T*)(1-A) where A is a nominal term for a rate-of-photosynthesis-dependent inhibition of starch degradation. Based on Figures 6 and 7, this mainly represents inhibition by Tre6P. A is set as 0.5 at standard growth light and 0.25 at light allowing 50% of photosynthesis in growth light; this parameterization is based on the ∼50% decrease in Tre6P when photosynthesis is halved (Supplemental Figure S5; Sulpice et al., 2014) and the response of the rate of starch degradation (∼50% decrease) to an approximately two-fold increase in Tre6P (Figure 6, see also Martins et al., 2013 for a similar dependence in the dark). Calculations were performed reiteratively at 2-h intervals. The amount of starch at the start of a time interval was corrected for (1) the amount of starch synthesized in the time interval and (2) the amount of starch that was degraded during the time interval to provide the initial value for S in the following time interval. The rate of net starch accumulation in a given time interval is calculated as *R*_s_ – *R*_d_ (Model 1) or *R*_s_ − *R*_d_* (Model 2). In panels D, H, and F where the response to a halving of light intensity was simulated, the rates of photosynthesis and starch synthesis were assumed to decrease in proportion with light intensity.

The impact of starch degradation in the light on the availability of C for growth and maintenance in a simulated twilight with or without starch degradation was modeled as in the legend of Supplemental Figures S7 and S10; Supplemental Calculations S3 and S4. Briefly, the rate of photosynthesis was set as proportional to momentary light intensity, the rate of starch synthesis was set as 33% (in short days) and 65% (in long days) of the rate of photosynthesis (Mengin et al., 2017). Starch content was summed cumulatively. Starch degradation rate in the dark was modeled as *R*_d_ = *S/T* where *S* is the amount of starch and *T* is the time until anticipated dawn (Scialdone et al., 2013). Starch degradation rate in the light is modeled in three ways: Model 0 (no starch degradation in the light), Model 1 (*R*_d_ = *S/T*), Model 2: *R*_d_* = (*S/T*)(1-A), where *A* is a nominal term for a rate of photosynthesis-dependent inhibition of starch degradation, set as proportional to light intensity, L. The calculations were applied iteratively over 1-h intervals. In Models 1 and 2, starch degradation in the light was allowed between ZT15 and ZT18 and between ZT0 and ZT3 (starch degradation outside this time interval was ignored as low compared to the rate of starch synthesis in high light). In Models 1 and 2, starch content in the light was corrected for starch lost by degradation. In all calculations, it was assumed that the clock is entrained to 3 h after first light, as is seen in plants growing in long days (Flis et al., 2016). The amount of C available for maintenance and growth was calculated by summing C directly available from photosynthesis (= photosynthesis – starch synthesis) and C provided by starch degradation.

### Statistical analyses

All statistical analysis were performed using Microsoft Excel (for details see Figure legends and Supplemental Data files).

## Supplemental data 

The following materials are available in the online version of this article.

**Supplemental Figure S1.** Comparison of net starch accumulation and maltose levels after transferring plants entrained in a 6- or 12-h photoperiod after transfer to low continuous light, or in plants growing in an 18-h photoperiod in low light (supplemental to Figure 1).

**Supplemental Figure S2.** Content of ^12^C and ^13^C starch during pulse labeling between ZT4 and ZT6 in plants growing in a 6-h photoperiod (supplemental to Figure 3A).

**Supplemental Figure S3.** Content of ^12^C and ^13^C starch during pulse labeling at different times in plants grown in an 18-h photoperiod and labeled with ^13^CO_2_ whilst leaving the plants at growth irradiance or after a sudden decrease in irradiance (supplemental to Figure 3B).

**Supplemental Figure S4.** Rate of photosynthesis after decreasing the light intensity from 105 to 65 µmol m^−2^ s^−1^ or decreasing the CO_2_ concentration from 380 to 95 ppm CO_2_ between ZT6 and ZT9, or between ZT10 and ZT13 (supplemental to Figure 5).

**Supplemental Figure S5.** Changes of sucrose and Tre6P after a decrease in light intensity (supplemental to Figure 4 and Supplemental Calculation S2; data are from the experiments shown in Figure 2).

**Supplemental Figure S6.** Content of ^12^C and ^13^C starch during pulse labeling before and after an induced increase in Tre6P (supplemental to Figure 7).

**Supplemental Figure S7.** Simulation of the impact of starch degradation in the light on the supply of C for growth in a simulated twilight without starch degradation in the light or with starch degradation occurring in the light (supplemental to Figure 8, Supplemental Figures S8 and S9, which experimentally test the simulations).

**Supplemental Figure S8.** Changes of starch, sucrose, and maltose and labeling patterns of Ala and Ser, glucose residues in the cell wall and Ala and Ser residues protein in a simulated dusk twilight in plants growing in long-day conditions (supplemental to Figure 8).

**Supplemental Figure S9.** Rates of cell wall and protein synthesis in a simulated dusk twilight in long-day conditions (this experiment is essentially a replicate of the one in Figure 8 and Supplemental Figure S8).

**Supplemental Figure S10.** Simulation of the response to a light drop between ZT4 and ZT6 in short-day-grown plants on C availability for growth in the dusk twilight (supplemental to Figure 9, which experimentally tests the simulation).

**Supplemental Figure 11.** Changes of starch, sucrose, and maltose and labeling patterns of Ala and Ser, glucose residues in the cell wall and Ala and Ser residues protein in a simulated dusk twilight in plants growing in short-day conditions (supplemental to Figure 9).

**Supplemental Figure S12.** ^13^CO_2_ labeling of starch in the first hours of the light period in long-day conditions (supplemental to Figure 10B).

**Supplemental Figure S13.** Starch degradation is negligible in the first hours of the light period in short-day conditions (supplemental to text in section “Starch degradation is negligible in the first hours of the light period in short day conditions”).

**Supplemental Figure S14.** Maltose, starch, ADPGlc, sucrose, glucose, and Glc6P content and *A*, after transferring plants to continuous light (all data except maltose are replotted from Fernandez et al., 2017).

**Supplemental Table S1.** List of experiments, design, and data sets.

**Supplemental Data Set 1.** Starch and metabolite levels after transfer of short day-grown plants to continuous low light (underlies Figure 1, A and C).

**Supplemental Data Set 2.** Starch and metabolite levels after transfer of long day-grown plants to continuous low light (underlies Figure 1, B and D).

**Supplemental Data Set 3.** Starch and maltose after transfer of short, neutral, and long day-grown plants to continuous low light—data compilation from this study and Fernandez et al. (2017) (underlies Supplemental Figure S1).

**Supplemental Data Set 4.** Starch and maltose levels and ^13^C labeling after subjecting short-day-grown plants to a light drop at ZT4–ZT6 (underlies Figures 2, A and 3, A; Supplemental Figures S2 and S4).

**Supplemental Data Set 5.** Starch and maltose levels after subjecting long-day-grown plants to a light drop at ZT4–ZT6 (underlies Figures 2, B and 3, B; Supplemental Figures S3, A, S5, C and D).

**Supplemental Data Set 6.** Starch labeling after a decrease in irradiance between ZT7–9 and ZT15–18 in long-day-grown plants left at growth light intensity (underlies Supplemental Figure S3B).

**Supplemental Data Set 7.** Response of starch content to a light drop between ZT6 and ZT12 in long-day-grown plants that were left at growth irradiance in the preceding days to ensure a substantial starch content at dawn, and then subjected to light drops at ZT6–ZT12 or ZT14–ZT20 (underlies Figure 4E).

**Supplemental Data Set 8.** Response of photosynthetic rate to a drop in light intensity or CO_2_ concentration after different times in the light (underlies Figure 5 and Supplemental Figure S4).

**Supplemental Data Set 9.** Levels of starch, maltose, and other metabolites in *TPS29.2* and *AlcR* control lines grown in a 12-h photoperiod and then transferred to continuous low light, induced with ethanol at ZT10 and harvested for analysis during the subjective night (ZT12–ZT24) (underlies Figure 6 and Supplemental Figures S5 and S6).

**Supplemental Data Set 10.** ^13^C labeling of starch, maltose and other metabolites in *TPS29.2* and *AlcR* control lines grown in a 12-h photoperiod and then transferred to continuous low light, induced with ethanol at ZT10; plants were pulsed with ^13^CO_2_ between ZT2 and ZT8, or between ZT14 and ZT20 (underlies Figure 7 and Supplemental Figure S7).

**Supplemental Data Set 11.** Rates of growth measured by ^13^CO_2_ enrichment in cell wall during a simulated twilight in long-day-grown plants (two separate experiments, underlying Figure 8 and Supplemental Figures S8 and S9).

**Supplemental Data Set 12.** Rates of growth measured by ^13^CO_2_ enrichment in cell wall during a simulated twilight in short-day-grown plants (underlies Figure 9 and Supplemental Figure S11).

**Supplemental Data Set 13.** Levels of starch and sugars in long day-grown plants maintained at growth irradiance until the day of the experiment, and then either left at growth irradiance or transferred at dawn to low irradiance: plants were harvested at dawn and at 2-h intervals in the first part of the light period (underlies Figure 10A).

**Supplemental Data Set 14.** Total starch content and ^13^C enrichment in starch in long day-grown plants maintained at growth irradiance throughout their growth and on the day of the experiment, and pulsed with ^13^CO_2_ for short periods in the first hours after dawn (two separate experiments are shown, that underlie Figure 10B and Supplemental Figure S12).

**Supplemental Data Set 15.** Total starch content and ^13^C enrichment in starch in wild-type Col-O and plants expressing a constitutively active form of ADP glucose pyrophosphorylase, grown in short-day conditions and pulsed with ^13^CO_2_ and harvested in the first hours after dawn (underlies Supplemental Figure S13, A–C).

**Supplemental Data Set 16.** Total starch content and ^13^C enrichment in starch wild-type Col-O and *dpe1* mutants (defective in *DISPROPORTIONATING ENZYME1* and therefore showing a starch excess phenotype at dawn even in short-day conditions) grown in short-day conditions and pulsed with ^13^CO_2_ and harvested in the first hours after dawn (underlies Supplemental Figure S13, D–F).

**Supplemental Data Set 17.** Maltose content in continuous light after transferring plants from a 12-h light/12-h dark cycle to continuous light (underlies Supplemental Figure S14).

**Supplemental Calculation S1.** Compiled changes in starch content in the first subjective night after transfer to continuous light (underlies Figure 4A).

**Supplemental Calculation S2.** Simulation of net starch content after transfer to continuous light or after light drops (underlies Figure 4).

**Supplemental Calculation S3.** Simulation of response of C availability for maintenance and growth during twilight in long-day-grown plants—comparison of three scenarios with no starch degradation in the light and with starch degradation simulated by the arithmetic division model and a modified arithmetic division model including feedback inhibition by Tre6P (underlies Figure S7).

**Supplemental Calculation S4.** Simulation of the impact of a sudden decrease in irradiance on C availability for maintenance and growth during twilight in short-day-grown plants (background to Figure 9 and Supplemental Figure S10).

## Supplementary Material

kiac162_Supplementary_Data

## Notes

A.M.S. and M.S. conceived the research with input from H.I. H.I., S.A., P.P., G.M.G., P.N., S.A., S.C.Z., A.S., R.F., A.R.F., and J.E.L. designed and performed much of the research and analyzed the data. M.S., A.M.S., and H.I. wrote the paper with contributions from the other authors.

The author responsible for distribution of materials integral to the findings presented in this article in accordance with the policy described in the Instructions for Authors (https://academic.oup.com/plphys/pages/general-instructions) is: Mark Stitt (mstitt@mpimp-golm.mpg.de).
