# Supplementary material for: Rising rates of starch degradation during daytime and trehalose 6-phosphate optimize carbon availability
Source: Plant Physiol. 2022 Apr 18;189(4):1976–2000. doi: 10.1093/plphys/kiac162 (PMC9342969; doi:10.1093/plphys/kiac162)

Supplemental Table S1: Description of experiments and data sets

Expt. ID	Experiment name	Sketch of the harvesting day	Photoperiod	Detailed experimental design	Used in	Data presented in
1	Temporal kinetics of starch accumulation when plants are grown in a 6-h photoperiod and transferred to continuous low light	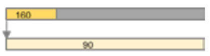	6-h light/18-h dark transfered to low continuous light	Plants were grown in a 6-h light/18-h dark cycle for 38 days with a light intensity of 160 µmol m^{-2} s^{-1} and then transferred at dawn to 90 µmol m^{-2} s^{-1} continuous light. The striped zone marks the subjective night.	Figure 1 A, C and Suppl. Fig.S1	Suppl. Data S1
2	Temporal kinetics of starch accumulation when plants are grown in an 18-h photoperiod.	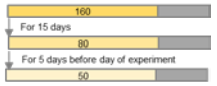	18-h light/6-h dark	Plants were grown in an 18-h light/6-h dark cycle for 15 days with a light intensity of 160 µmol m^{-2} s^{-1}, switched to 80 µmol m^{-2} s^{-1} for five days to reduce starch levels at dawn, and then harvested during an 18-h light period at 50 µmol m^{-2} s^{-1}.	Figure 1B, D and Suppl. Fig.S1	Suppl. Data S2
3	Response of starch content and maltose to a decrease in light intensity at different times in the light period in plants growing in a short photoperiod.	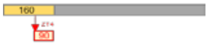	6-h light/18-h dark	Plants were grown in a 6-h light/18-h dark cycle at a light intensity of 160 µmol m^{-2} s^{-1} for 38 days. On the 39th day after sowing, plants were left at 160 µmol m^{-2} s^{-1} for 6 h (black circles), or were illuminated at 160 µmol m^{-2} s^{-1} until ZT4 and then transferred to 90 µmol m^{-2} s^{-1} from ZT4 to ZT6 (red circles). $^{13}CO_2$ was supplied between ZT4 and ZT6.	Figure 2 A-B, 3A, Suppl. Fig.S2, S5	Suppl. Data S4
4	Response of starch content and maltose to a decrease in light intensity at different times in the light period in plants growing in a long photoperiod	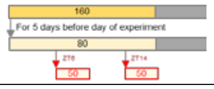	18-h light/6-h dark	Plants were grown in an 18-h light/6-h dark cycle for 15 days at a light intensity of 160 µmol m^{-2} s^{-1}, transferred to 80 µmol m^{-2} s^{-1} for 5 days to reduce dawn starch levels, then on the 21st day after sowing were left at 80 µmol m^{-2} s^{-1} (black symbols) or were initially illuminated at 80 µmol m^{-2} s^{-1} and then transferred to 50 µmol m^{-2} s^{-1} between ZT6 and ZT10, or between ZT14 and ZT18 (red circles).	Figure 2C-D, 3B, Suppl. Fig.S3, S5	Suppl. Data S5
6	$^{13}CO_2$ labelling of starch during a decrease in irradiance at different times in the light period in an 18-h photoperiod	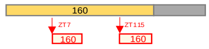	18-h light/6-h dark	In the middle of the light period and before dusk in long day-grown plants. Plants were grown in an 18-h light, 6-h dark cycle at 160 µmol m^{-2} s^{-1} for 20 days, and on the 21st day were supplied with $^{13}CO_2$ between ZT7 and ZT9 or between ZT15 and ZT18.	Figure S3B	Suppl. Data S6
7	Response of starch content to a decrease in light intensity at ZT6 and ZT14 in plants grown in an 18-h photoperiod at 160 umol µmol m^{-2} s^{-1}	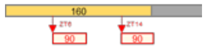	18-h light/6-h dark	In the middle of the light period and before dusk in long day-grown plants. Plants were grown in an 18-h light, 6-h dark cycle at 160 µmol m^{-2} s^{-1} for 15 days followed by 5 days at 90 µmol m^{-2} s^{-1}, and on the 21st day were supplied with $^{13}CO_2$ between ZT7 and ZT9 or between ZT15 and ZT18.	Figure 4 E	Suppl. Data S7
8	Response of starch content to a drop in light intensity or CO_2 concentration after different times in the light.	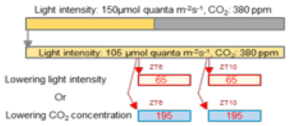	12-h light/12-h dark	Plants were grown in a 12 h photoperiod at 150 µmol quanta m^{-2} s^{-1} and 380 ppm CO_2 for 24 days. On the day of the experiment the plants were illuminated at 105 µmol m^{-2} s^{-1}, 380 ppm CO_2. Some were transferred to lower light (65 µmol m^{-2} s^{-1}) or lower CO_2 (195 ppm CO_2) at ZT6 and harvested at ZT9. Others were transferred to lower light (65 µmol m^{-2} s^{-1}) or lower CO_2 (195 ppm CO_2) at ZT10 and left in these conditions until harvest at ZT13. Control plants remained in 105 µmol m^{-2} s^{-1} and 380 ppm CO_2 until ZT13.	Figure 5, Suppl. Fig.S4	Suppl. Data S8
9	Impact of an induced increase in Tre6P on starch content and metabolite levels in the light	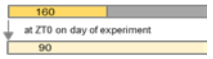	12-h light/12-h dark and transferred to low continuous light	*TPS29.2* and *AlcR* plants (black symbols, *TPS29.2*; grey symbols, *AlcR*) were grown in a 12-h light/12-h dark cycle at a light intensity of 160 µmol m^{-2} s^{-1} for 21 days and then transferred to continuous light at 90 µmol m^{-2} s^{-1}. The plants were sprayed with 2% v/v ethanol at ZT10 and samples were harvested at 3-h intervals, starting at ZT12.	Figure 6	Suppl. Data S9
10	Impact of an induced increase in Tre6P on 13CO2 labelling of starch and metabolites in the light.	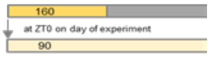	12-h light/12-h dark and transferred to low continuous light	The experiment was conducted as in Fig. 6, spraying with 2% v/v ethanol at ZT10 to induce an increase in Tre6P (see Fig. 6 for the changes of Tre6P, total starch content and other metabolites). $^{13}CO_2$ was supplied at ambient concentration (420 ppm) between ZT2 and ZT8 (before the induced increase in Tre6P) and between ZT14 and ZT20 (after the induced increase in Tre6P).	Figure 7, Suppl. Fig.S6	Suppl. Data S10
11	Rates of cell wall synthesis and protein synthesis in a simulated evening twilight in plants growing in long day conditions, measured by $^{13}CO_2$ labelling.	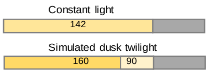	18-h light/6-h dark or with simulated twilight between ZT14 to ZT18	Plants were grown in 18 h light/6 h dark cycles with a light intensity of 142 µmol m^{-2} s^{-1} throughout the light period (black) or with a light intensity of 160 µmol m^{-2} s^{-1} from ZT0 to ZT14 and a light intensity of 90 µmol m^{-2} s^{-1} from ZT14 to ZT18 (simulated dusk twilight, red). Estimated rates (fractional increase per day) of cell wall synthesis	Figure 8, Suppl. Fig.S8	Suppl. Data S11A
12	Rates of cell wall synthesis and protein synthesis in a simulated evening twilight in plants growing in long day conditions, measured by $^{13}CO_2$ labelling.	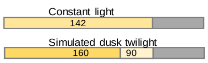	18-h light/6-h dark or with simulated twilight between ZT14 to ZT18	Plants were grown in 18 h light/6 h dark cycles with a light intensity of 142 µmol m^{-2} s^{-1} throughout the light period (black) or with a light intensity of 160 µmol m^{-2} s^{-1} from ZT0 to ZT14 and a light intensity of 90 µmol m^{-2} s^{-1} from ZT14 to ZT18 (simulated dusk twilight, red). Estimated rates (fractional increase per day) of cell wall synthesis	Suppl. Fig S9	Suppl. Data S11B
13	Rates of cell wall and protein synthesis in a simulated evening twilight in short day conditions, measured by $^{13}CO_2$ labelling.	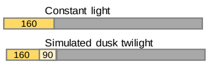	6-h light/18-h dark or with simulated twilight between ZT4 to ZT6	Plants were grown in a 6 h light/18 h dark cycle at a light intensity of 160 µmol m^{-2} s^{-1} throughout the light period (step light regime, black) or with a light intensity of 160 µmol m^{-2} s^{-1} from ZT0 to ZT4 and a light intensity of 90 µmol m^{-2} s^{-1} from ZT4 to ZT6 (simulated dusk twilight, red). Estimated rates (fractional increase per day) of cell wall synthesis.	Figure 9, Suppl. Fig.S11	Suppl. Data S12
14	Starch degradation in the first hours of the light period in long day conditions.	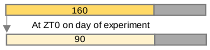	18-h light/6-h dark	Plants were grown in in a 18-h light/6- dark cycle at 160 µmol m^{-2} s^{-1} for 21 days and then either left at growth irradiance (black symbols) or transferred to 90 µmol m^{-2} s^{-1} (red symbols). Plants were harvested just after dawn and at 2 h intervals for the first part of the light period.	Figure 10 A	Suppl. Data S13
15	Starch degradation in the first hours of the light period in long day conditions measured by 13CO2 labelling		18-h light/6-h dark	Plants were grown for 21 days in a 18-h light/6-h dark cycle at 160 µmol m^{-2} s^{-1} and on the 22nd day were supplied with $^{13}CO_2$ between ZT0.25 and ZT1.25, or between ZT0.25 and ZT2.25 or between ZT7 and ZT9. Plants were harvested at the start and end of each labeling interval.	Figure 10 B, Suppl. Fig.S12	Suppl. Data S14
16	Starch degradation is negligible in the first hours of the light period in short day conditions (*adp1*)		8-h light/16-h dark	Plants were grown in 8-h light/16-h dark cycle with 340µmol $m^{-2}s^{-1}$ light intensity, and pulsed with $^{13}CO_2$ between ZT0.25 and ZT2.25.	Suppl. Fig S13A-C	Suppl. Data S15
17	Starch degradation is negligible in the first hours of the light period in short day conditions (*dpe1*)		8-h light/16-h dark	Plants were grown in 8-h light/16-h dark cycle with 340µmol $m^{-2}s^{-1}$ light intensity, and pulsed with $^{13}CO_2$ between ZT0.25 and ZT2.25.	Suppl. Fig S13D-F	Suppl. Data S16
18	Changes in intermediates of starch and maltose in plants under continuous light (Fernandez et al., 2017)	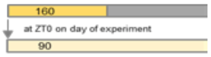	12-h light/12-h dark and transfered to low continuous light	Plants were grown in a 12-h light/12-h dark cycle at a light intensity of 160 µmol m^{-2} s^{-1} for 21 days and then transferred to continuous light at 90 µmol m^{-2} s^{-1}.	Suppl. Fig. S1, S14	Suppl. Data S17

Supplemental Fig. S1 Comparison of net starch accumulation and maltose levels (after transferring plants entrained in a 6-h or 12-h photoperiod to low continuous light, or in plants growing in an 18-h photoperiod in low light (supplemental to Fig. 1).

(A) Starch
(B) Maltose

The data for the 6-h (gray symbol) and 18-h (red symbol) photoperiod are from Fig. 1, and the data for the 12-h (black symbol) photoperiod are from Fernandez et al. (2017). The error bars are 95% confidence limit with replicates (n = 5 – 6, except ZT0 when n =3).

Underlying data are provided in Supplemental Datasets S1, S2 and S3.

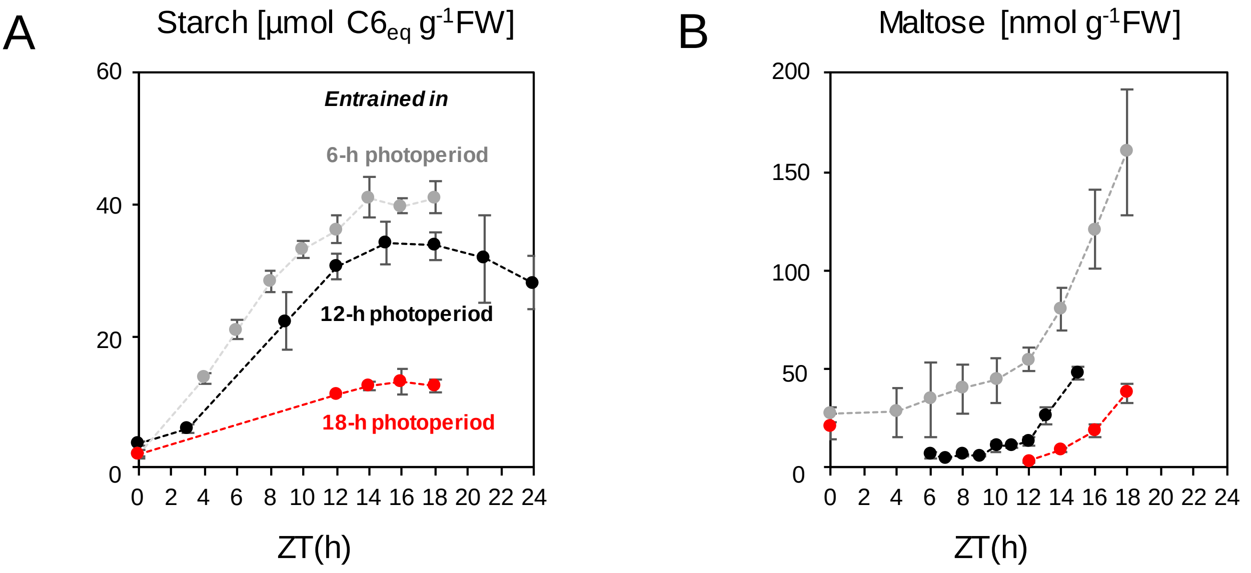

Experimental design

Entrained to a 6-h photoperiod and transferred to lower continuous light on the day of the experiment

Entrained to a 12-h photoperiod and transferred to lower continuous light on the day of the experiment (from Fernandez et al., 2017)

Entrained to an 18-h photoperiod and transferred to lower light before the experiment to ensure starch was exhausted by dawn, and to even lower light on the day of the experiment

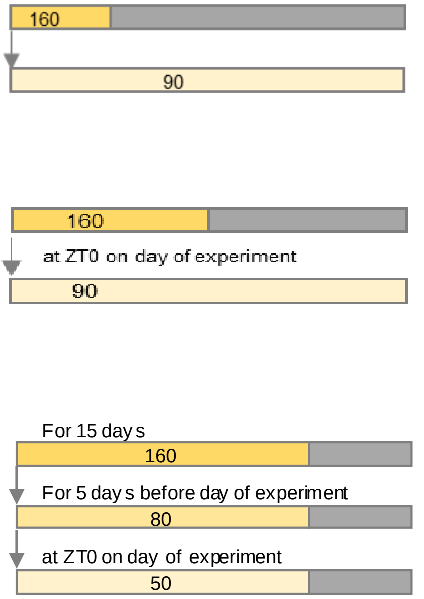

Supplemental Fig. S2 Content of ^{12}C and ^{13}C starch during pulse labeling between ZT4 and ZT6 in plants growing in a 6-h photoperiod (supplemental to Fig 3A).

Plants were grown in 6-h light/18-h dark cycles for 38 days with a light intensity of 160 µmol m^{-2} s^{-1} for 38 days. On the 39th day, plants were illuminated at 160 µmol m^{-2} s^{-1} until ZT4 and were then transferred to 90 µmol m^{-2} s^{-1} from ZT 4 to ZT6 (starch level shown in Fig 3A). $^{13}CO_2$ was supplied between ZT4 and ZT6.

The bar diagram shows the amount of ^{12}C starch (light grey bars) and ^{13}C starch (white bars) at ZT4 and ZT6. Total starch is given by the sum of the grey and white areas. The table provides the values used for the figure. The error bars are 95% confidence limit with replicates (6 biological replicates, each containing 5 individual rosettes except ZT6 where n = 8).

The data were used to calculate the change in ^{12}C and ^{13}C starch content shown in Fig. 3A.

Underlying data are provided in Supplemental Dataset S4.

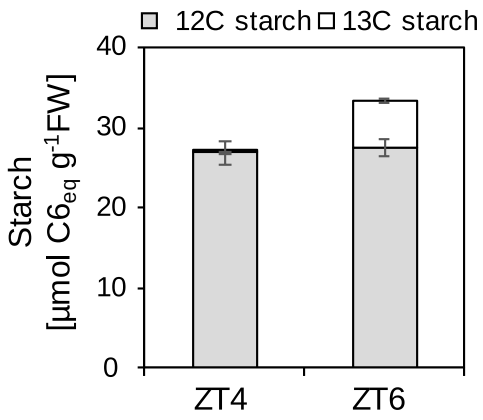

ZT (hour)	Starch [µmol $C6_{eq}$ g^{-1}FW]		
	Total	13C	12C
4	27.02 ± 1.46	0.02 ± 0.01	27.00 ± 1.47
6	33.52 ± 1.58	5.97 ± 0.30	27.55 ± 1.11

Mean ± 95% confidence interval

Experimental design

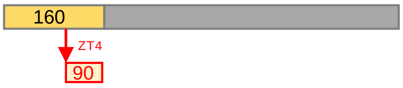

Supplemental Fig. S3. Content of 12C and 13C starch during pulse labeling at different times in plants grown in an 18-h photoperiod and labelled with 13CO2 whilst leaving the plants at growth irradiance or after a sudden decrease in irradiance (supplemental to Fig 3B).

(A) Plants were grown for 15 days in 18 h light, 6 h dark cycles at 160 µmol m^{-2} s^{-1} and 5 days at 80 µmol m^{-2} s^{-1}. At the 21st day, illumination was again provided at 80 µmol m^{-2} s^{-1}, and one set of plants was transferred to lower light (50 µmol m^{-2} s^{-1} between ZT 6 until ZT0 and another set between ZT14 until ZT18. $^{13}CO_2$ was supplied between ZT6 and ZT10 or between ZT14 and ZT18.

Plants were harvested before the decrease in irradiance and provision of $^{13}CO_2$, and at the end of the 4-h treatment in low irradiance with $^{13}CO_2$.

The bar diagrams show the amount of ^{12}C starch (light grey bars) and ^{13}C starch (white bars) before and after feeding of $^{13}CO_2$ for the time intervals indicated in the panel. Total starch is the sum of the grey and white areas. The tables provide the values used for the figures. All values are means ± 95% confidence interval of measurements (4-5 biological replicates, each containing 5 individual rosettes)

The data were used to calculate the change in ^{12}C and ^{13}C starch content shown in Fig. 3B.

The underlying data are provided in Supplemental Data S5.

A

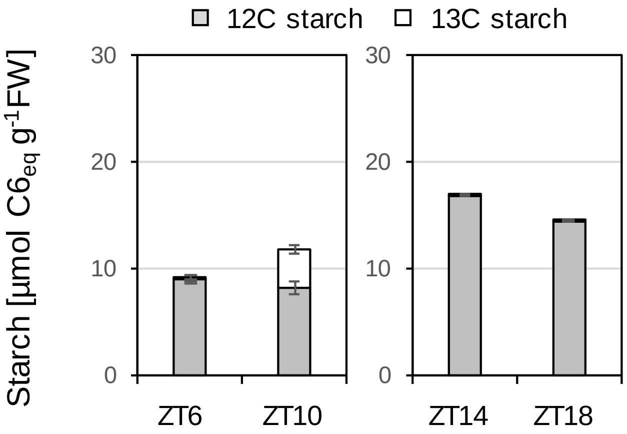

ZT (hour)	Starch [µmol C6eq g^{-1}FW] Total	13C	12C
6	9.1 ± 0.3	0.0 ± 0.0	9.0 ± 0.3
10	12.4 ± 0.5	3.6 ± 0.4	8.3 ± 0.6
14	17.7 ± 1.0	0.2 ± 0.0	16.9 ± 1.6
18	15.7 ± 0.9	0.2 ± 0.0	14.4 ± 0.9

Mean ± 95% confidence interval

Experimental design

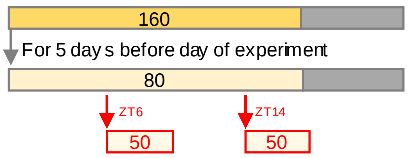

Panel B is on the next page

Supplemental Fig. S3 (continued)

(B) This experiment differs form that of panel A in that plants were left at a higher irradiance on the days before the experiment and on the day of the experiment, and $^{13}CO_2$ was supplied at growth irradiance. Plants were grown for 21 days in 18 h light, 6 h dark cycles at 160 µmol m^{-2} s^{-1} On the day of the experiment, $^{13}CO_2$ was supplied in growth conditions between ZT7 and ZT9 or between ZT15 and ZT18.. Plants were harvested before the decrease in irradiance and provision of $^{13}CO_2$, and at the end of the 4-h treatment in low irradiance with $^{13}CO_2$.

The bar diagrams show the amount of ^{12}C starch (light grey bars) and ^{13}C starch (white bars) before and after feeding of $^{13}CO_2$ for the time intervals indicated in the panel. Total starch is the sum of the grey and white areas. The tables provide the values used for the figures. All values are means ± 95% confidence interval of measurements (4-5 biological replicates, each containing 5 individual rosettes)-

The underlying data are provided in Supplemental Data S6.

This experiment shows in long-day grown plants, starch degradation speeds up with time in the light at growth light intensity. Comparison of this experiment with that of Fig. 3 and Supplemental Fig S3A shows that starch degradation speeds up less at growth light intensity than when the light intensity is decreased at ZT15.

B

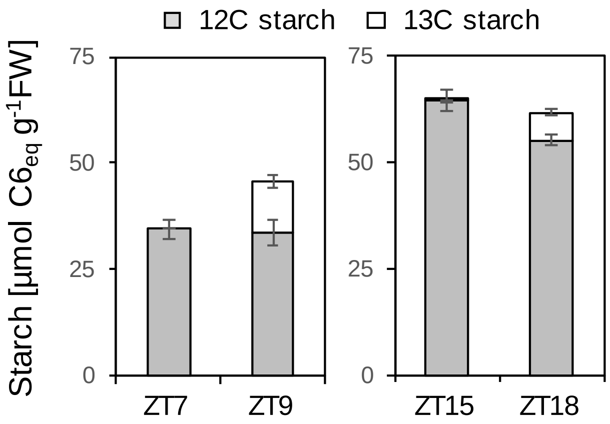

ZT (hour)	Starch [µmol C6eq g^{-1}FW]		
	Total	13C	12C
7	34.6 ± 2.2	0.0 ± NA	34.6 ± 2.2
9	45.4 ± 3.2	12.0 ± 1.4	33.8 ± 3.0
15	64.7 ± 2.4	0.0 ± 0.0	64.7 ± 2.4
18	63.3 ± 3.1	6.5 ± 0.8	55.3 ± 1.1

Mean ± 95% confidence interval

Calculated rates of ^{12}C and ^{13}C starch accumulation

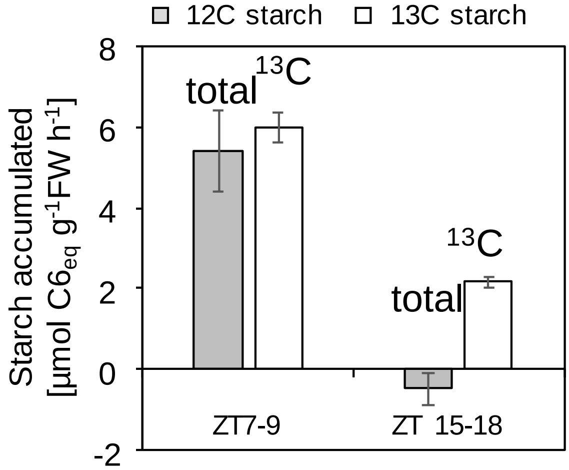

Experimental design

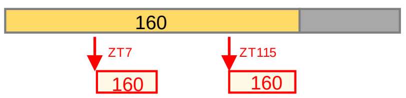

Supplemental Fig. S4. Rate of photosynthesis after decreasing the light intensity from 105 to 65 µmol m^{-2} s^{-1} or decreasing the CO_2 concentration from 380 to 95 ppm CO_2 between ZT6 and ZT9, or between ZT10 and ZT13 (supplemental to Fig. 5).

Photosynthesis was measured at ~30 min intervals in control plants at 105 µmol m^{-2} s^{-1} and 380 ppm CO_2, and in plants after transfer to low light intensity or to low CO_2. The data are the mean of 4 replicate measurements. The experiment was conducted as described in the legend to Fig. 5.

These supplemental data provide evidence that the decrease in light intensity and CO_2 concentration led to approximately the same inhibition of photosynthesis, and that this inhibition was similar after a decrease in light intensity and CO_2 at ZT6 and after a decrease in light intensity and CO_2 at ZT10. Results are given as mean ± SD (n = 3-4).

The underlying data are shown in Suppl. Dataset S8.

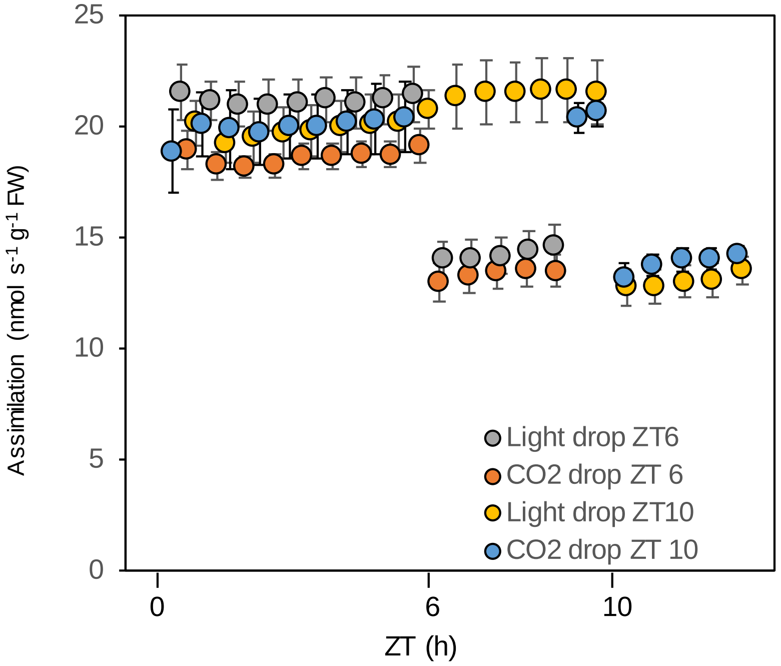

Experimental design

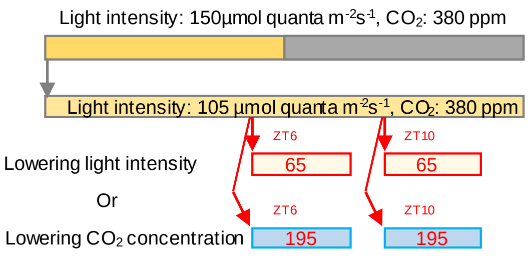

Supplemental Fig. S5. Changes of sucrose and Tre6P after a decrease in light intensity (Supplemental to Fig 4G-H and Supplemental Calculation S2; data are from the same experiments as those shown in Fig. 2).

(A,B) Plants were grown 6-h /18-h light/dark cycle with light intensity of 160 µmol m^{-2} s^{-1} for 38 days. On the 39th day after sowing, plants were left at 160 µmol m^{-2} s^{-1} for 6 h, or were illuminated 160 µmol m^{-2} s^{-1} until ZT4 and were then transferred to 90 µmol m^{-2} s^{-1} from ZT4 to ZT6 (sucrose and Tre6P levels after the light drop are shown as red symbols). Error bars are 95% confidence limit with replicates (n <5 replicate samples except ZT0, n=3)

(C,D) Plants were grown 18-h /6-h light/dark cycle for 15 days with light intensity of 160 µmol m^{-2} s^{-1}, switched to 80 µmol m^{-2} s^{-1} for 5 days to reduce starch levels at dawn. On the 21st day after sowing, plants wee either left at 80 µmol m^{-2} s^{-1}, or were initially illuminated at 80 µmol m^{-2} s^{-1} and then transferred to 50 µmol m^{-2} s^{-1} for 4 h from ZT6to ZT10, or from ZT14 to ZT18 (sucrose and Tre6P levels after the light drop are shown as red symbols). Error bars are 95% confidence limit with replicates (n <5 except ZT0, n=3)

(A,C) Sucrose

(B, D) Tre6P

Underlying data and analyses of further metabolites are provided in Supplemental Data S4 (short day grown plants) and S5 (long day-grown plants)

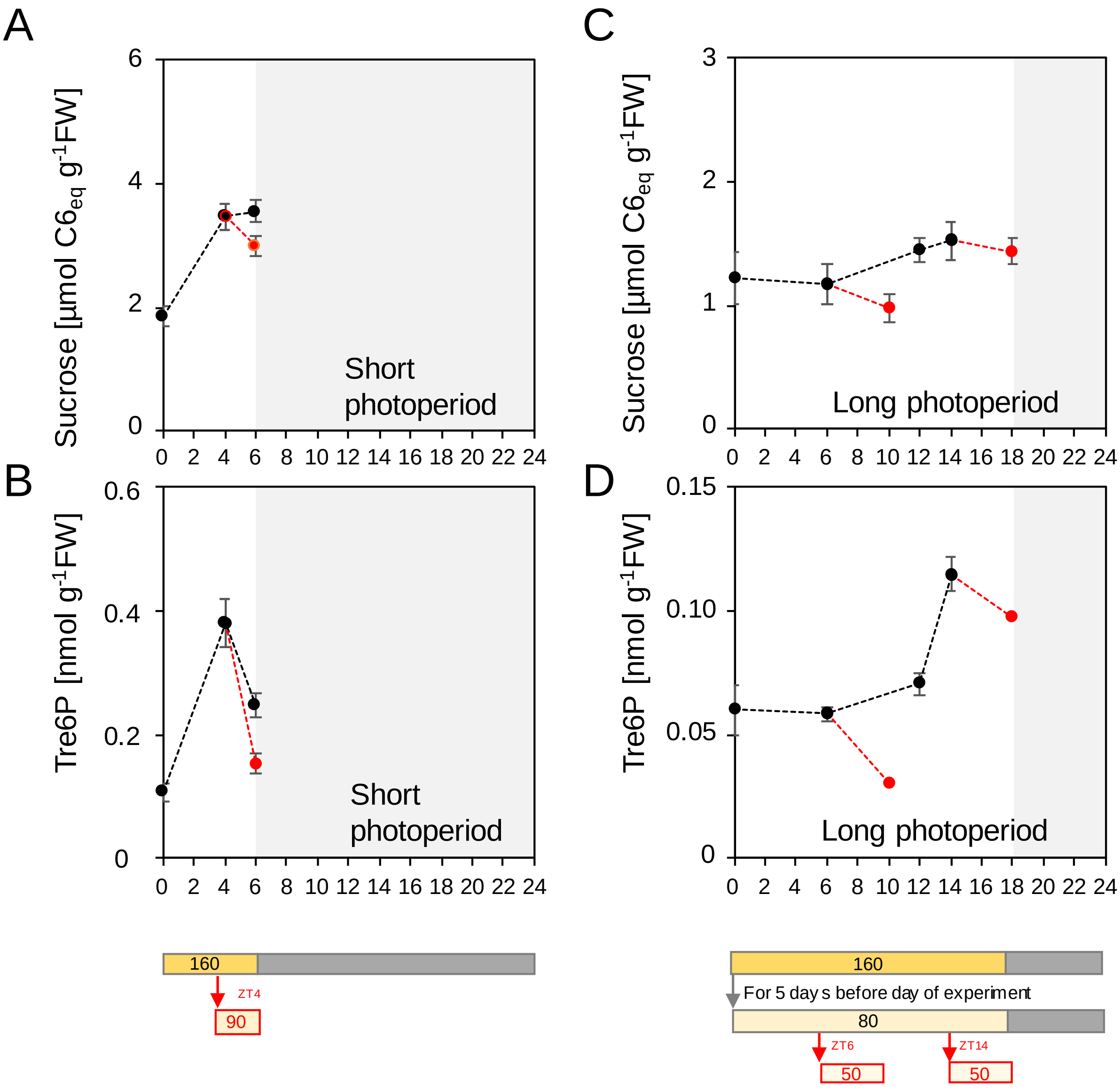

Supplemental Fig. S6 Content of ^{12}C and ^{13}C starch during pulse labeling before and after an induced increase in Tre6P (supplemental to Fig 7).

TPS29.2 (black symbols) and *AlcR* (gray symbols) were grown in a 12-h light/12-h dark cycle at a light intensity of 160 µmol m^{-2} s^{-1} for 21 days and then transferred at dawn to continuous light at 90 µmol m^{-2} s^{-1}. All of the plants were sprayed with 2% v/v ethanol at ZT10 to induce an increase in Tre6P (see Fig. 5 for the changes of Tre6P, total starch content and other metabolites). $^{13}CO_2$ was supplied at ambient concentration (420 ppm) between ZT2 and ZT8 (before the induced increase in Tre6P) and between ZT14 and ZT20 (after the induced increase in Tre6P).

The bar diagrams show the amount of ^{12}C starch (light grey bars) and the amount of ^{13}C starch (white bars) before and after feeding $^{13}CO_2$ over the time intervals indicated under the panels. Total starch is given by the sum of the grey and white areas. The tables to the right provide the values used for the figures.

All values are means ± 95% confidence interval of measurements (5 biological replicates, each containing 5 individual rosettes)

Underlying data are provided in Supplemental Dataset S10.

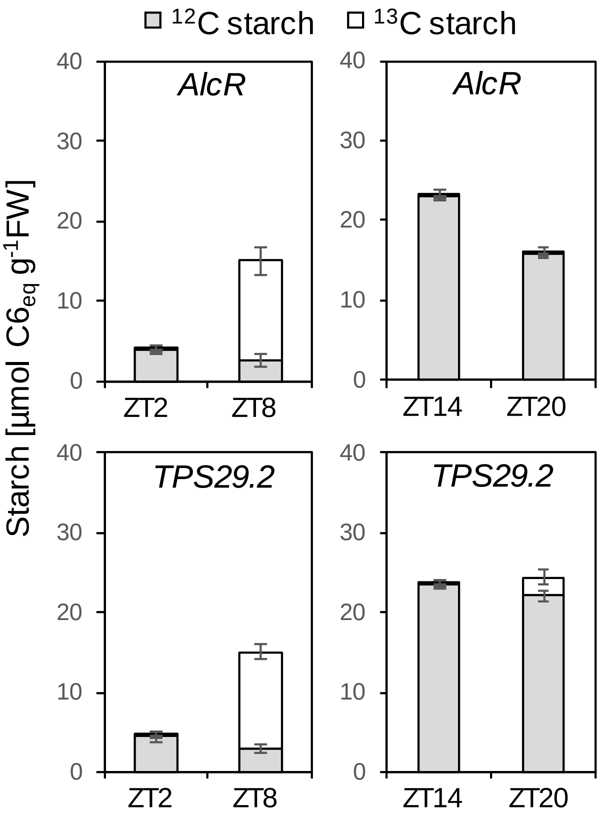

ZT (hour)	*AlcR*: Starch [µmol $C6_{eq}$ g^{-1}FW]		
	Total	^{13}C	^{12}C
2	3.93 ± 0.59	0.00 ± 0.00	3.93 ± 0.59
8	15.13 ± 1.54	12.51 ± 1.72	2.62 ± 0.74
14	23.17 ± 1.99	0.01 ± 0.01	23.16 ± 1.99
20	15.91 ± 1.64	0.00 ± 0.01	15.91 ± 1.64

Mean ± 95% confidence interval

ZT (hour)	*TPS29.2:* Starch [µmol $C6_{eq}$ g^{-1}FW]		
	Total	^{13}C	^{12}C
2	4.47 ± 0.58	0.00 ± 0.00	4.47 ± 0.58
8	15.12 ± 0.87	12.13 ± 1.05	2.98 ± 0.63
14	23.50 ± 2.70	0.01 ± 0.01	23.49 ± 2.70
20	24.47 ± 2.85	2.36 ± 0.77	22.11 ± 2.16

Mean ± 95% confidence interval

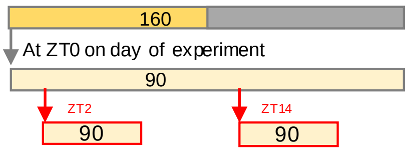

Supplemental Fig. S7 Schematic representation of the impact of starch degradation in the light on the availability of C for growth and maintenance in a simulated twilight with or without starch degradation in the light (supplemental to Fig. 8 and Supplemental Figs. S8 and S10, which experimentally test the simulation).

The simulation is for plants growing in an 18-h photoperiod, with a gradual rise in irradiance between ZT0 and ZT3 and a gradual decrease in light intensity between ZT15 and ZT18.

(A) **Scheme of the underlying metabolic model**. In the simplest case (step light regime) or a gradual change in intensity, the availability of C for growth in the light is equal to the amount of fixed C (set as 1) minus the amount of C allocated to starch (set as 0.33, based on empirical data in Sulpice et al., 2014; Mengin et al., 2017) plus (in simulations where this occurs) the modelled rate of starch degradation in the light. The availability of C for growth and maintenance at night in a given time interval is equivalent to the rate of starch degradation. The approaches used to estimate the rate of starch degradation are explained in the legend to panel C. The model assumes full use of all fixed C within a 24-h cycle and assumes negligible C is stored in the roots. Rates are given as a fraction of the rate of photosynthesis.

(B) **Photosynthesis and starch synthesis.** The rate of photosynthetic C fixation and the rate of starch synthesis in a given one-hour time interval are set as proportional to the average light intensity in the preceding time interval. Starch synthesis is set as 33% of C fixation (see legend to panel A). The amount of C available directly from photosynthesis for growth and maintenance in the light is 0.67 x photosynthesis (not shown).

(C) **Starch degradation.** The starch degradation rate in the dark, R, is modelled according to the skeletal arithmetic division equation (Scialdone et al., 2013), such that R_d = S/T where S is the amount of starch and T is the time until anticipated dawn. It is assumed that the clock is entrained to 3 h after first light, as is seen in plants growing in a long photoperiod (Flis et al., 2016). The rate of starch degradation in the light is modelled in three ways:
Model 0: (dotted line **……**): there is no starch degradation in the light.
Model 1: (dashed line **------**): the rate of starch degradation in the light is set by the skeletal arithmetic division equation as R_d = S/T, with degradation occurring in the light from ZT15 onwards, and between ZT0 and ZT3. For simplicity, starch degradation outside this time interval is ignored as low compared to the rate of starch synthesis in high light.
Model 2: (solid line ──): the rate of starch degradation is set by a modified skeletal arithmetic division equation in which the rate is decreased in proportion to the light intensity, as R_d* = (S/T) (1 - L), where L is light intensity, proportional to photosynthesis rate, A. As in model 1, starch degradation in the light is allowed only between ZT15 and ZT18 and between ZT0 and ZT3
The symbols R_d and R_d* are used to indicate that these terms are analogous those in the models in Fig. 4.

(D) **Amount of C available for maintenance and growth**. This is calculated by summing C available directly from photosynthesis (photosynthesis – starch synthesis, panel A) and C available from starch degradation (panel B). This is shown for the three scenarios presented in panel C.

The underlying calculations are provided in Supplemental Calculation S3.

Supplemental Fig. S7 (continued)

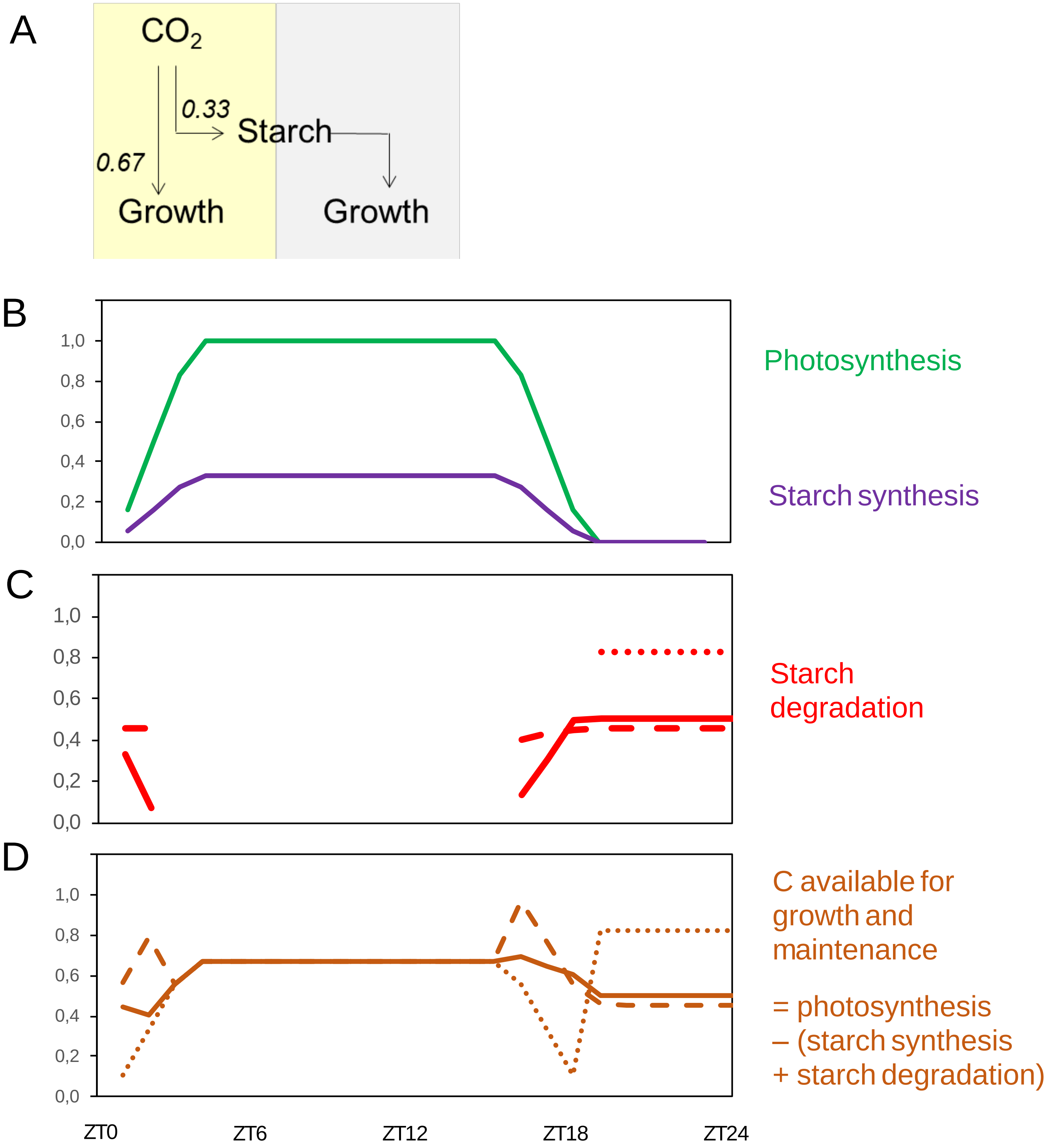

Model 0 (…...) No starch degradation in light

Model 1 (------) Starch degradation in light between ZT0 and ZT3 and Z15 and ZT18 as predicted by the arithmetic division model

Model 2 (——) Starch degradation in light between ZT0 and ZT3 and Z15 and ZT18, as predicted by the arithmetic division model but decreased in proportion to irradiance.

Supplemental Fig. S8. Changes of starch, sucrose and maltose and labeling patterns of free Ala and Ser, glucose residues in the cell wall and Ala and Ser residues in protein in a simulated dusk twilight in plants growing in long days (Supplemental to Fig. 8).

Plants were grown in a 18-h light/6-h dark cycle with a light intensity of 142 µmol m^{-2} s^{-1} throughout the light period (black circles) or with a light intensity of 160 µmol m^{-2} s^{-1} from ZT0 to ZT14 and a light intensity of 90 µmol m^{-2} s^{-1} from ZT14 to ZT18 (red circles, simulated dusk twilight). Red arrows indicate the time at which irradiance was decreased. On the 21st day, plants were labelled with $^{13}CO_2$ for 24 h, starting just before ZT0, and samples were harvested every 4 h.

Light intensity (A), total levels of (B) starch, (C) sucrose and (D) maltose. ^{13}C enrichment in (E) Ala and (F) Ser in the soluble fraction. ^{13}C enrichment in (G) glucose in the cell wall fraction and (H) Ala and (I) Ser in the protein fraction.

All values are given as mean and 95% confidence limit (n = 5).
Underlying data are provided in Supplemental Dataset S11A.

These data were used to calculate the rate of glucose incorporation into the cell wall and the rate of protein synthesis (see Fig. 8)

A similar experiment is shown in Supplemental Fig. S9

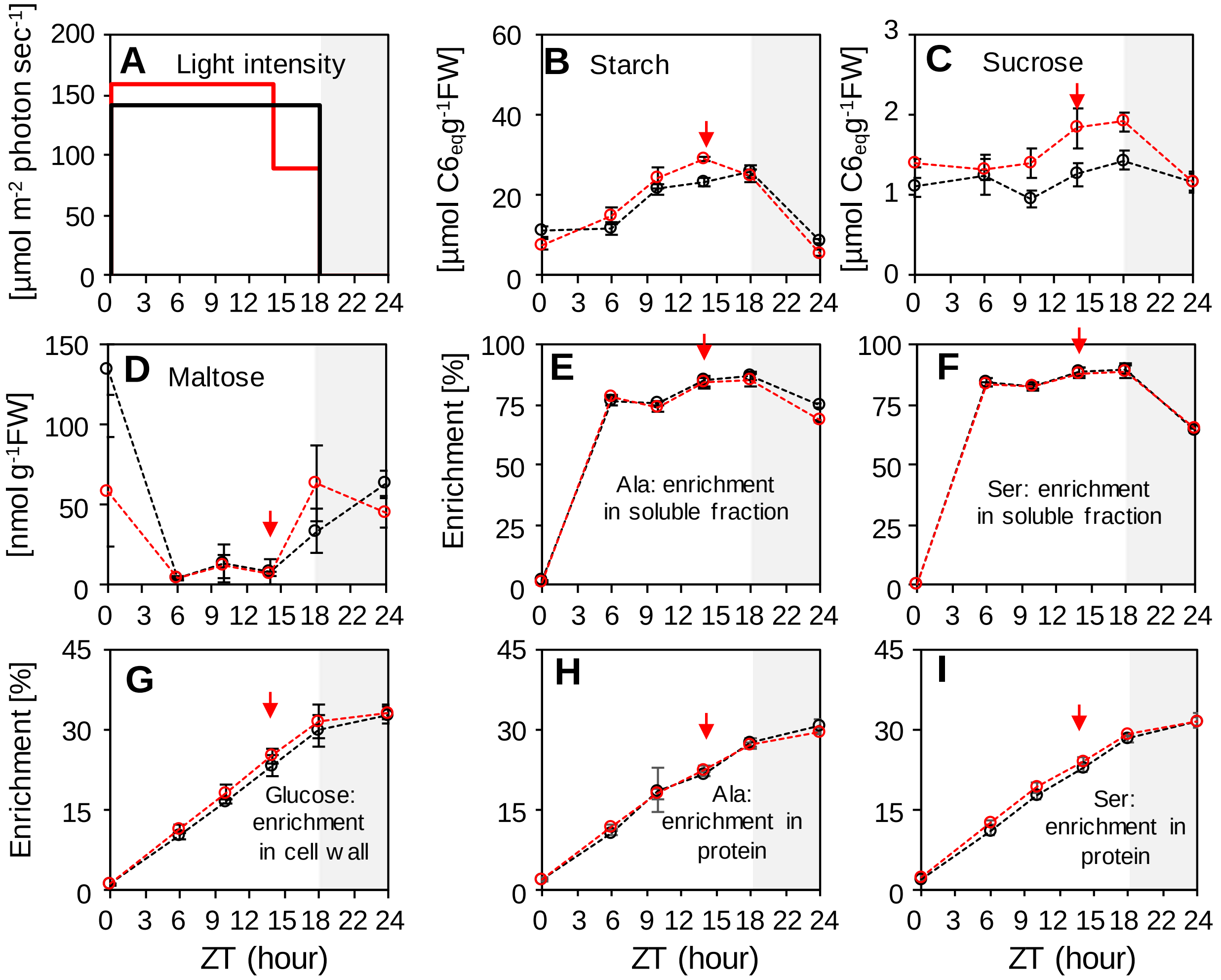

Supplemental Fig. S9 Rates of protein synthesis and cell wall synthesis in a simulated dusk twilight in long day conditions (this experiment is essentially a replicate of the one in Fig. 8).

The plants were grown in a 18-h light 6-h dark cycle with a light intensity of 142 µmol m^{-2} s^{-1} throughout the light period (black circles) or with a light intensity of 160 µmol m^{-2} s^{-1} from ZT0 to ZT14 and a light intensity of 90 µmol m^{-2} s^{-1} from ZT14 to ZT18 (red circles = simulated dusk twilight). Red arrows in panels A-I indicate the time at which irradiance was decreased. On the 21st day, plants were labelled with $^{13}CO_2$ for 24 h, starting just before ZT0 and samples were harvested every 4 h. Light intensity (A). Total levels of (B) starch, (C) sucrose and (D) maltose. ^{13}C enrichment in (E) Ala and (F) Ser in the soluble fraction. ^{13}C enrichment in (G) glucose in the cell wall fraction and (H) Ala and (I) Ser in the protein fraction. All values are given a mean and 95% confidence limit (n = 5). Rates (% increase per day) of cell wall synthesis (J) or protein synthesis estimated using Ala (K) or Ser (L) in the constant light regime (black bar) and simulated dusk twilight (red bar). Rates of protein synthesis were estimated as the increase in enrichment in a given time interval in Ala or Ser in protein, corrected for incomplete enrichment in free Ala or Ser (see Ishihara et al., 2015; 2017).The error bars for flux into cell wall and protein were calculated using the standard error of the mean for Gaussian error propagation (Birge, 1939; Ku, 1966). Note that precursor pools are either labelled directly from fixed $^{13}CO_2$ or, if starch or other reserves are being remobilised, from ^{13}C incorporated into these reserves earlier in the 24-h cycle. Underlying data are given in Supplemental Dataset S11B.

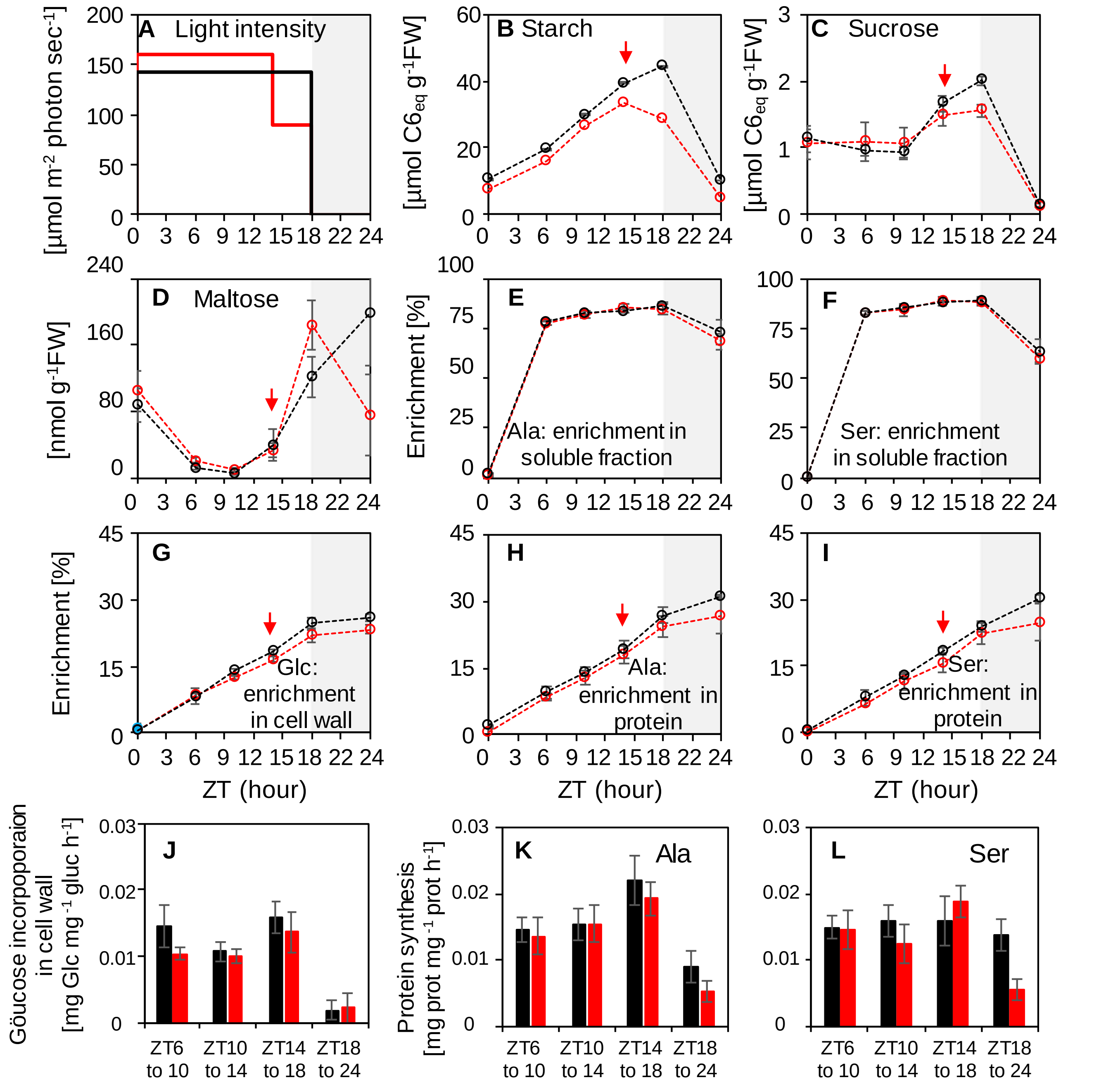

Supplemental Fig S10. Simulation of the impact of a light drop between ZT4 and ZT6 in short day–grown plants on C availability for growth in the dusk twilight (Supplemental to Fig 9, which tests the simulation).

C available for growth was estimated from the rates of photosynthesis and starch accumulation or mobilisation (the term growth includes maintenance and C respired at night). C availability in the light was estimated as the net rate of photosynthesis (set at 1 in standard light) minus the proportion of fixed C allocated to starch plus (in the case that starch degradation occurs in the light) the rate of starch degradation. C availability at night was set as the rate of starch degradation. The rate of starch degradation in any given condition and time was estimated using the skeletal arithmetic division equation, R_d = S/T, where R_d = the rate of starch degradation, S = the amount of starch at that time and T = the time to the next dusk (see Supplemental Calculation S4

(**A) Plants growing in short days.** The upper panel shows the underlying model for C allocation: 65% of fixed C is allocated to starch and 35% to growth (see Mengin et al., 2017). The lower panels show the response to dusk twilight, simulated by decreasing irradiance in the last 2 h before dusk to a level that results in a 50% or a 75% decrease in photosynthesis. C availability for growth was estimated using two scenarios: i) no starch degradation in the light (blue), ii) starch is degraded in the light at a rate predicted by the skeletal arithmetic division equation (orange). The simulations predict that starch degradation can buffer C availability against at twilight in short day-grown plants, even though the absolute rates of starch degradation are not yet high (see Figs. 1-3). Thus, predicted C availability falls proportionately with the drop in photosynthesis when there is no starch degradation in the light, and this fall is almost completely or partly (after a 50% and 75% drop in photosynthetic rate, respectively) reverted in simulations where starch is degraded in the light.

B) Plants growing in short days, but with C allocation to starch decreased to the level found in long day-gown plants. This simulation explores the importance of the higher allocation of photosynthate to starch observed in short-day plants for their ability to buffer C availability for growth in the dawn twilight. The underlying metabolic model assumes that only 35% of fixed C is allocated to starch (corresponding to the value observed in long day-grown plants (see Mengin et al., 2017). As in panel A, twilight was simulated as a decrease in light intensity that leads to either a 50% or a 75% decrease in photosynthesis. The predicted availability of C falls proportionately with the drop in photosynthesis when there is no starch degradation in the light. This is only slightly attenuated when starch degradation in allowed in the light. It might be noted that this simulation also explores how long-day grown plants would respond to a sudden drop in light intensity early in the light period.

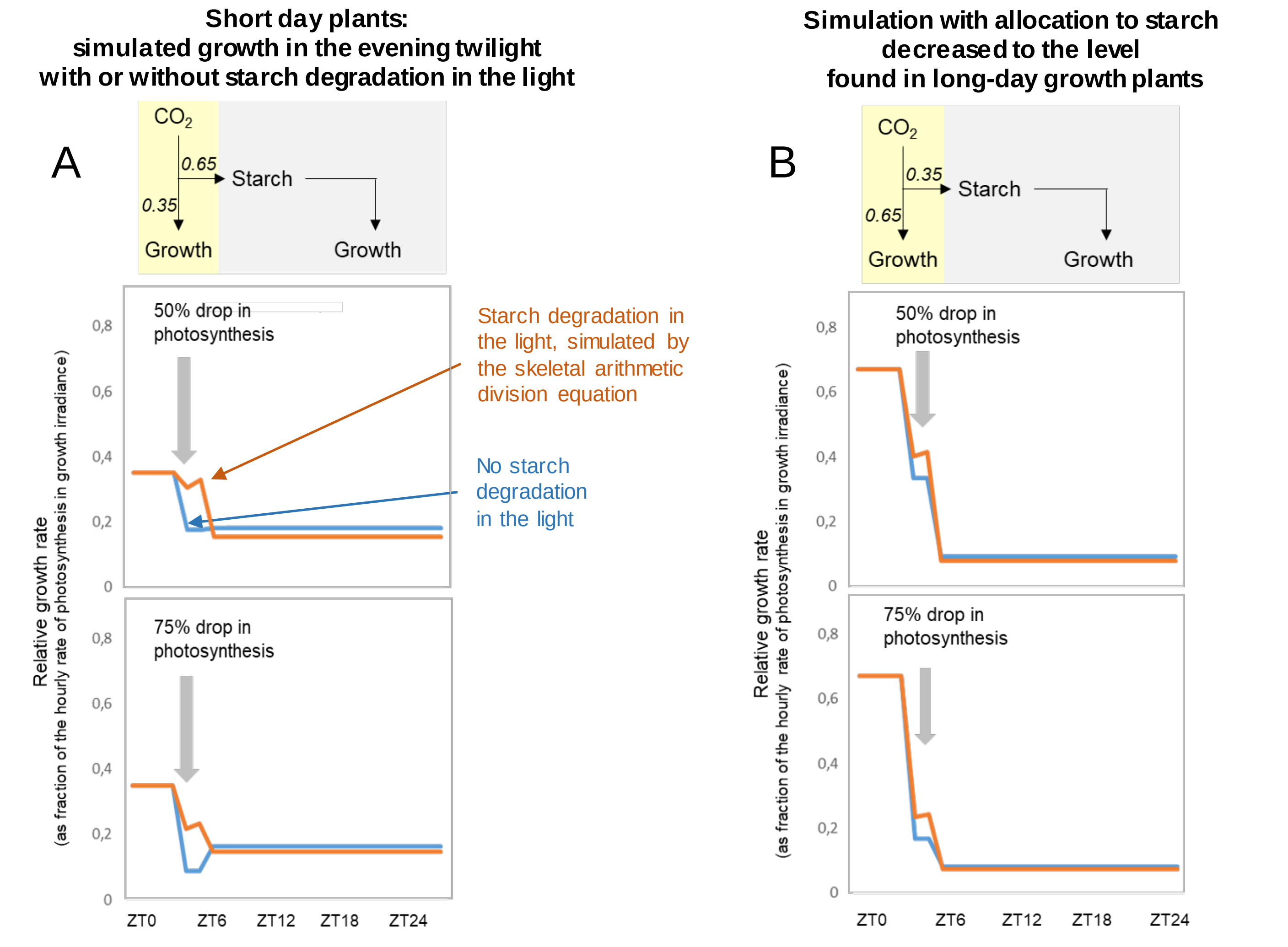

Supplemental Fig. S11. Changes of starch, sucrose and maltose and labeling patterns of free Ala and Ser, glucose residues in the cell wall and Ala and Ser residues in protein in a simulated dusk twilight in plants growing in short day conditions (Supplemental to Fig. 9).

Plants were grown in a 6-h light/18-h dark cycle at a light intensity of 160 µmol m^{-2} s^{-1} throughout the light period (step light regime, black circles) or with a light intensity of 160 µmol m^{-2} s^{-1} from ZT0 to ZT4 and a light intensity of 90 µmol m^{-2} s^{-1} from ZT4 to ZT6 (simulated dusk twilight, red circles). Red arrows indicate the time at which irradiance was decreased. On the 28th day, plants were labelled with $^{13}CO_2$ for 24 h, starting just before ZT0 and samples were harvested 30 minutes, 1, 4, 6, and 24 h after starting of the pulse.

Light intensity (A). Total levels of (B) starch, (C) sucrose and (D) maltose. ^{13}C enrichment in (E) Ala and (F) Ser in the soluble fraction. ^{13}C enrichment in (G) glucose in the cell wall fraction and (H) Ala and (I) Ser in the cell wall fraction.

All values are given a mean and 95% confidence limit (n = 5).
Underlying data are provided in Supplemental Dataset S12.

These data were used to calculate the rate of glucose incorporation into the cell wall and the rate of protein synthesis (see Fig. 9)

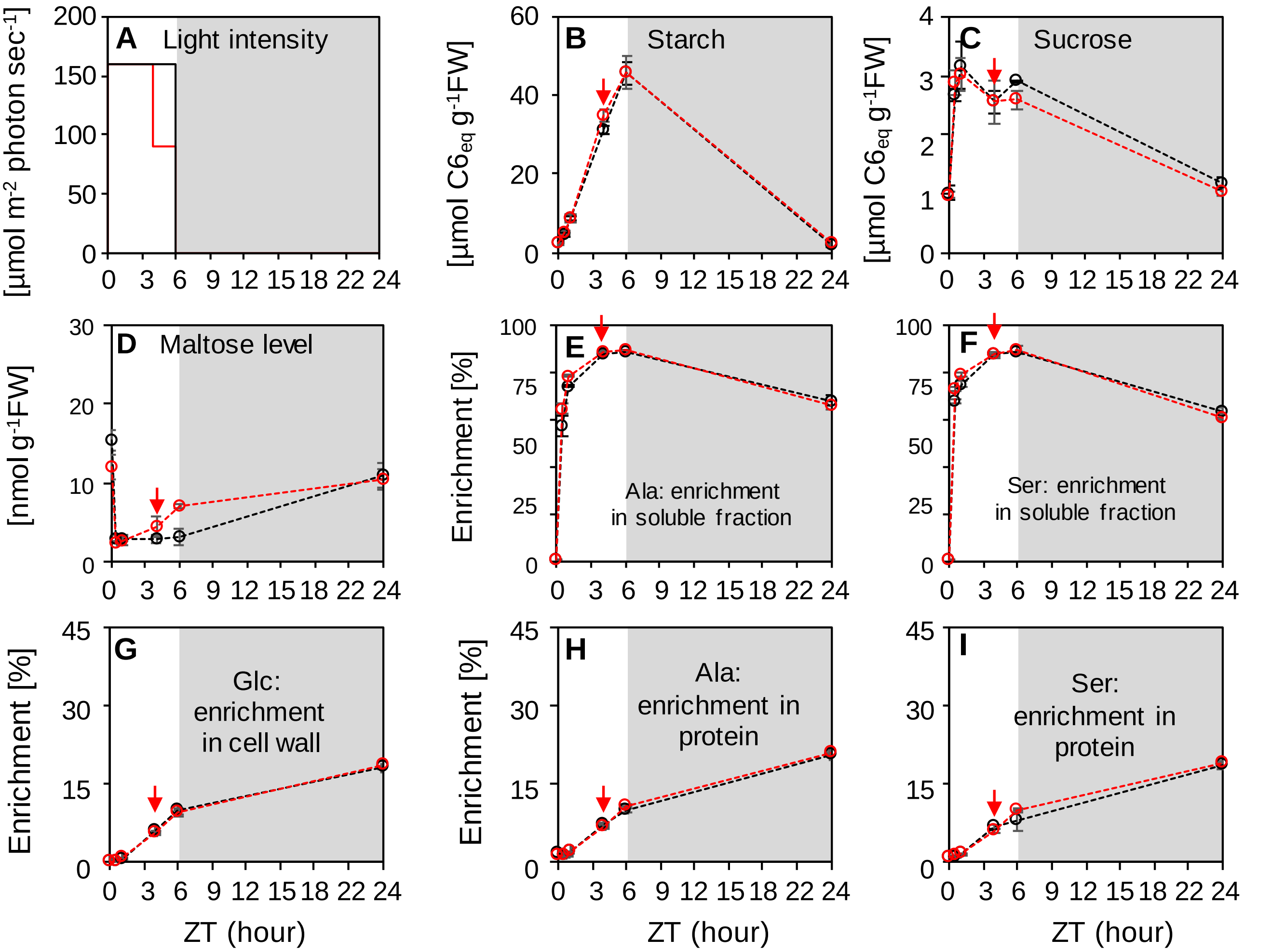

Supplemental Fig. S12. $^{13}CO_2$ labelling of starch in in the first hours of the light period in long day conditions (supplemental to Fig. 10B).

Plants were grown for 21 days in 18 h light, 6 h dark cycle at 160 µmol m^{-2} s^{-1} and on the 22nd day were supplied with $^{13}CO_2$ between ZT0.25 and ZT1.25, or between ZT0.25 and ZT2.25 or between ZT7 and ZT9. Plants were harvested at the start and end of each labeling interval, starch isolated, hydrolyzed to glucose and analyzed by GC-MS.

The bar diagrams show the amount of ^{12}C starch (light grey bars) and ^{13}C starch (white bars) before and after feeding $^{13}CO_2$ for the time intervals indicated under the figures. Total starch is the sum of the grey and white areas.
The tables provide the values used for the figures. Data were used to calculate the change in ^{12}C and ^{13}C starch content shown in Fig. 10B.

All values are means ± 95% confidence interval of measurements (4-5 biological replicates, each containing 5 individual rosettes).

The underlying data are provided in Supplemental Dataset S14

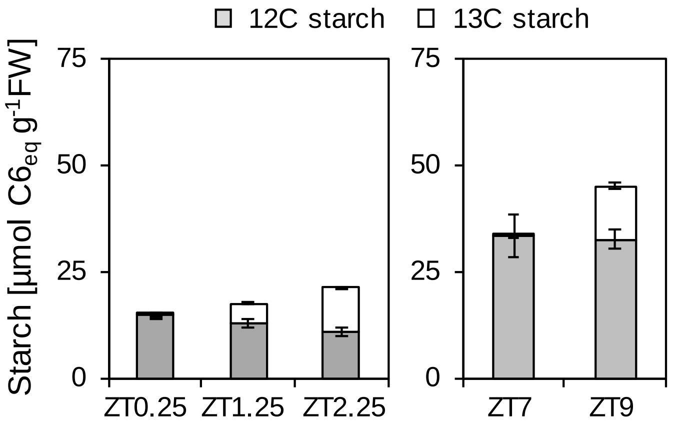

ZT (hour)	Starch [µmol $C6_{eq}$ g^{-1}FW]		
	Total	13C	12C
0.25	14.9 ± 0.8	0.0 ± 0.0	14.9 ± 0.8
1.25	17.7 ± 1.2	4.8 ± 0.4	12.9 ± 1.1
2.25	21.4 ± 0.8	10.7 ± 0.2	10.8 ± 1.0
7	33.6 ± 5.0	0.0 ± 0.0	33.6 ± 5.0
9	45.2 ± 2.6	12.6 ± 0.7	32.6 ± 2.3

Mean ± 95% confidence interval

Experimental design

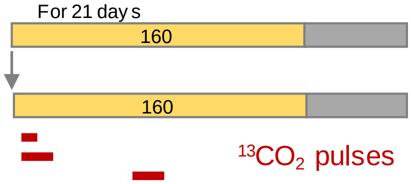

Supplemental Fig. S13. Starch degradation is negligible in the first hours of the light period in short day conditions (supplemental to text in section 'There is no starch degradation in the early part of the light period in short day conditions).

All plants were grown in 8-h light/16-h dark cycle with 340µmol $m^{-2}s^{-1}$ light intensity and pulsed with $^{13}CO_2$ between ZT0.25 and ZT2.25.

(A,D) Total amount of starch
(B,E) Amount of starch labeled with ^{12}C and ^{13}C, shown with gray and white bars respectively.
(C,F) Estimated overall net change in starch content (grey bar) and rate of ^{13}C incorporation in starch respectively.

(A,B,C) *adp1* complemented *adp1/ASP1$_{WT}$* (wild-type, Haedrich et al., 2011). Results are the mean ± 95% confidence limit (A and B). (n=7 except ZT4, n=4,) The error bars were calculated using the standard error of the mean for Gaussian error propagation in C.
(D,E,F) starch excess mutant *dpe1*. The results are the mean ± 95% confidence limit (D and E). (n=7 except ZT 0 and 4, n=5). The error bars were calculated using the standard error of the mean for Gaussian error propagation in F.

The underlying data are provided in Supplemental Datasets S15 and S16.

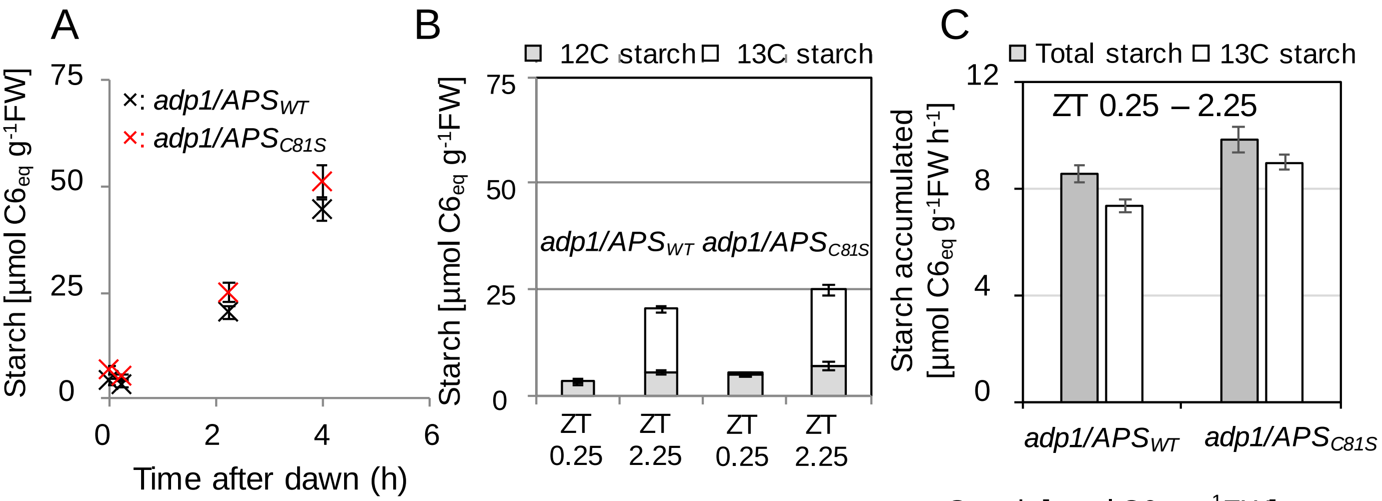

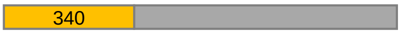

Genotype	ZT (hour)	Starch [µmol C6$_{eq}$ g^{-1}FW] Total	13C	12C
adp1/APS1$_{WT}$	0.25	3.42 ± 0.66	0.00 ± 0.00	3.42 ± 0.66
adp1/APS1$_{WT}$	2.25	20.44 ± 1.07	14.72 ± 0.95	5.71 ± 0.53
adp1/APS1$_{C81S}$	0.25	5.25 ± 0.52	0.00 ± 0.00	5.25 ± 0.52
adp1/APS1$_{C81S}$	2.25	24.93 ± 1.77	17.96 ± 1.12	6.97 ± 1.08

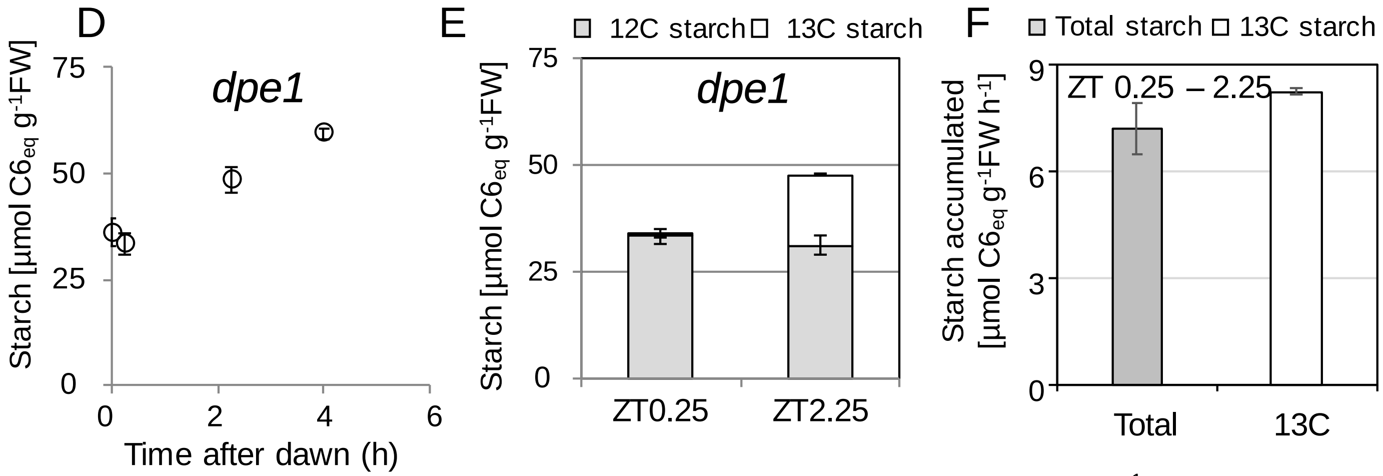

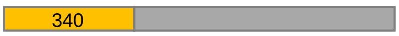

ZT (hour)	Starch [µmol C6$_{eq}$ g^{-1}FW] Total	13C	12C
0.25	33.39 ± 1.89	0.01 ± 0.01	33.38 ± 1.89
2.25	47.77 ± 2.23	16.47 ± 0.34	31.31 ± 2.13

Supplemental Fig. S14. Maltose, starch, ADPGlc, sucrose, glucose and Glc6P content and photosynthesis rate after transferring plants to continuous light (all data except maltose are replotted from Fernandez et al., 2017).

Plants were grown for 21 days at 160 µmol m^{-2} s^{-1}, then transferred at dawn to continuous light at 90 µmol m^{-2} s^{-1}. Plants were harvested and assayed for metabolites at intervals over 33 h. The data except maltose were obtained from Fernandez et al. (2017). Maltose was measured for this publication in the same material as used in Fernandez et al. (2017). The striped zone represents the subjective night. Values are means ± 95% confidence interval (n ≥ 4, except maltose at ZT0 when n=2)

The underlying data are provided in Supplemental Datafile S17

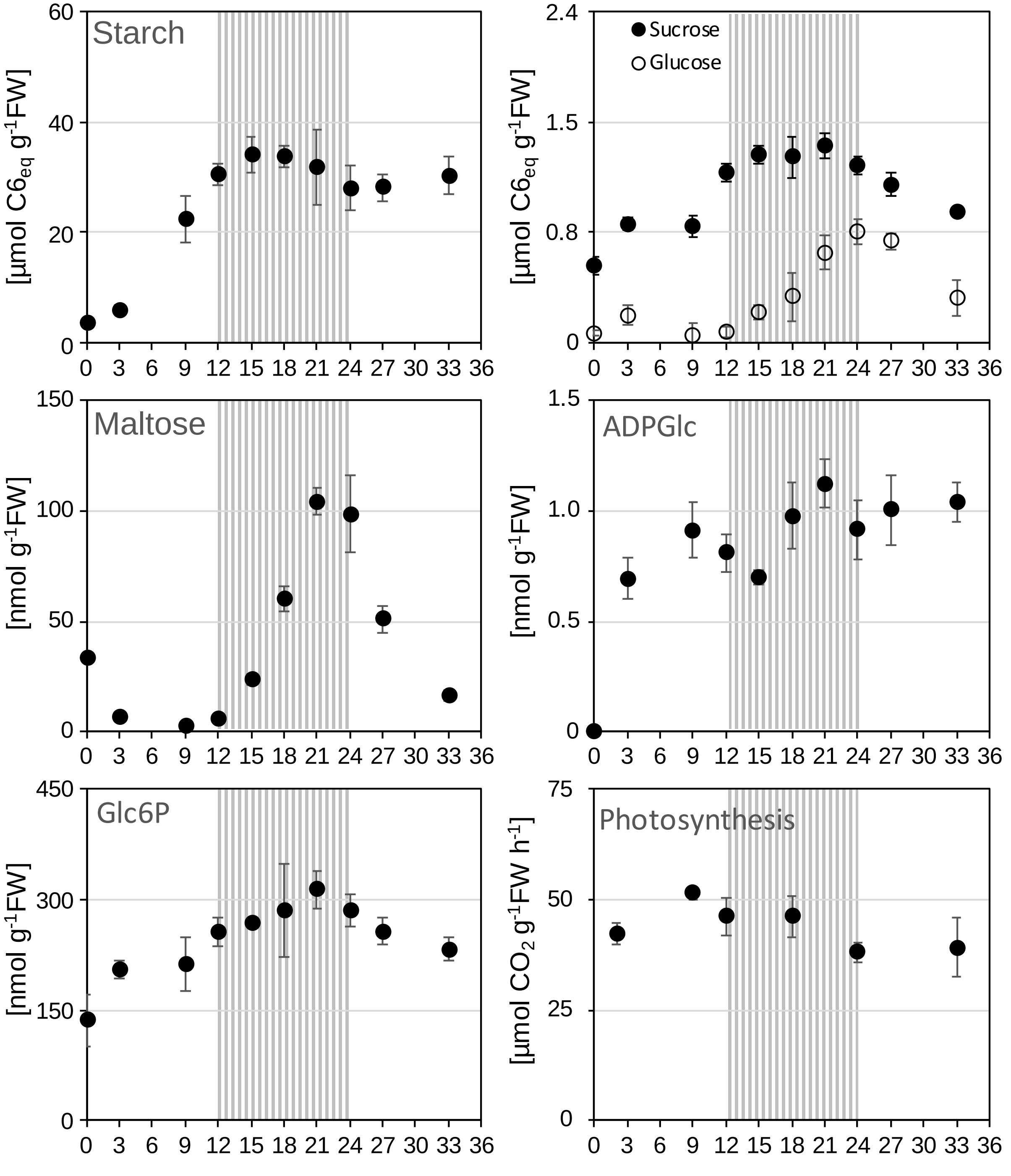

Supplement: kiac162_Supplementary_Data [file kiac162_supplementary_data.zip › kiac162_Supplementary_Data/Ishihara et al Supplemental Material_Suppl Table S1 and Suppl. Figs S1S14.pdf]
